# Ecological constraints on highly evolvable olfactory receptor genes and morphology in neotropical bats

**DOI:** 10.1111/evo.14591

**Published:** 2022-08-30

**Authors:** Laurel R. Yohe, Matteo Fabbri, Daniela Lee, Kalina T. J. Davies, Thomas P. Yohe, Miluska K. R. Sánchez, Edgardo M. Rengifo, Ronald P. Hall, Gregory Mutumi, Brandon P. Hedrick, Alexa Sadier, Nancy B. Simmons, Karen E. Sears, Elizabeth Dumont, Stephen J. Rossiter, Bhart‐Anjan S. Bhullar, Liliana M. Dávalos

**Affiliations:** ^1^ Department of Earth and Planetary Sciences Yale University New Haven Connecticut 06511 USA; ^2^ Department of Ecology and Evolution Stony Brook University Stony Brook New York 11794 USA; ^3^ Deaprtment of Bioinformatics and Genomics University of North Carolina at Charlotte Charlotte North Carolina 28223 USA; ^4^ North Carolina Research Campus Kannapolis North Carolina 28081 USA; ^5^ Negaunee Integrative Research Center Field Museum of Natural History Chicago Illinois 60605 USA; ^6^ Harvard School of Medicine Cambridge Massachusetts 02115 USA; ^7^ School of Biological and Behavioural Sciences Queen Mary University of London London E1 4NS United Kingdom; ^8^ Independent Software Engineer Centerville OH USA; ^9^ Escuela Profesional de Ciencias Biológicas Universidad Nacional de Piura Piura 20004 Peru; ^10^ Programa de Pós‐Graduação Interunidades em Ecologia Aplicada, Escola Superior de Agricultura ‘Luiz de Queiroz’, Centro de Energia Nuclear na Agricultura Universidade de São Paulo Piracicaba 13416‐970 Brazil; ^11^ Centro de Investigación Biodiversidad Sostenible (BioS) Lima 15073 Peru; ^12^ School of Natural Sciences University of California, Merced Merced California 95344 USA; ^13^ Department of Biomedical Sciences Cornell University Ithaca New York 14853 USA; ^14^ Department of Ecology and Evolutionary Biology University of California, Los Angeles Los Angeles California 90095 USA; ^15^ Department of Mammalogy American Museum of Natural History New York New York 10024 USA; ^16^ Yale Peabody Museum of Natural History Yale University New Haven Connecticut 06511 USA; ^17^ Center for Inter‐Disciplinary Environmental Research Stony Brook University Stony Brook New York 11794 USA

**Keywords:** Chemosensation, evolvability, gene family, morphology, olfaction

## Abstract

Although evolvability of genes and traits may promote specialization during species diversification, how ecology subsequently restricts such variation remains unclear. Chemosensation requires animals to decipher a complex chemical background to locate fitness‐related resources, and thus the underlying genomic architecture and morphology must cope with constant exposure to a changing odorant landscape; detecting adaptation amidst extensive chemosensory diversity is an open challenge. In phyllostomid bats, an ecologically diverse clade that evolved plant visiting from a presumed insectivorous ancestor, the evolution of novel food detection mechanisms is suggested to be a key innovation, as plant‐visiting species rely strongly on olfaction, supplementarily using echolocation. If this is true, exceptional variation in underlying olfactory genes and phenotypes may have preceded dietary diversification. We compared *olfactory receptor* (*OR*) genes sequenced from olfactory epithelium transcriptomes and olfactory epithelium surface area of bats with differing diets. Surprisingly, although *OR* evolution rates were quite variable and generally high, they are largely independent of diet. Olfactory epithelial surface area, however, is relatively larger in plant‐visiting bats and there is an inverse relationship between *OR* evolution rates and surface area. Relatively larger surface areas suggest greater reliance on olfactory detection and stronger constraint on maintaining an already diverse *OR* repertoire. Instead of the typical case in which specialization and elaboration are coupled with rapid diversification of associated genes, here the relevant genes are already evolving so quickly that increased reliance on smell has led to stabilizing selection, presumably to maintain the ability to consistently discriminate among specific odorants—a potential ecological constraint on sensory evolution.

Many cellular pathways are under strong constraint to maintain function: the fixation of potentially lethal mutations can disrupt core functions, and thus natural selection more frequently removes than favors novel mutations. However, systems that are more exploratory in nature in that they must interact with an ever‐changing environmental space (e.g., adaptive immunity, host‐detection avoidance [Graves et al. 2013; Merrikh and Merrikh 2018]) may possess a greater capacity to evolve, that is, increased evolvability. With increased variation, there is more opportunity to generate phenotypic diversity and interact with new stimuli, facilitating the occupation of novel adaptive zones (Simpson 1953; Feiner et al. 2021). At the same time, rampant diversification is expected to come under constraint (i.e., purifying or stabilizing selection) from ecological limits (Kirschner and Gerhart 1998). New variation may enable exploration of novel niche space, but once a shift has occurred into a new adaptive zone, selection may fine‐tune genes and phenotypes to optimize performance within that environment. As a result, specialization will occur, and novel constraints will maintain that specialized system in the new zone. Although previous work has demonstrated how increased heritable variation may promote evolvability (Graves et al. 2013), how ecology restricts this variation is less well understood.

The mammalian olfactory system offers an excellent framework for evaluating links between genomic and phenotypic evolvability and ecological diversity. Here, the genetic and morphological components of scent detection are both highly variable and interactive, resulting in a complex environmental chemical space directly relevant to fitness (Yohe and Brand 2018). In contrast to host‐pathogen immunity and infection dynamics, in which there is strong selection to either infect or avoid infection, the fitness consequences of the vast functional repertoire of the olfactory system may be less dire on average. *Olfactory receptor* genes (*OR*s) encode G‐protein‐coupled receptor proteins that combinatorially respond to chemical bouquets, relaying signals critical to finding food, avoiding predators, attracting mates, avoiding noxious chemicals, identifying conspecifics, and caring for offspring (Doty 1986; Kurian et al. 2021). The *OR* multigene family is both the largest and among the fastest‐evolving protein‐coding gene families in the mammalian genome (Niimura 2012; Niimura et al. 2014). The highly evolvable nature in this family extends throughout tetrapods (Yohe et al. 2020b). The patterns observed in the *OR* multigene family are generated via a birth‐death evolutionary process of tandem gene duplication, leading to highly clustered unstable genomic regions (Nei and Rooney 2005). Gene duplication generates new substrates for selectable variation: so long as negative dosage effects are minimal, new gene copies are released from selective constraints and can accumulate novel mutations through which the gene can diversify or lose function (Yohe et al. 2019b). Although the *OR* multigene family is composed of hundreds of genes, they can be classified into subfamilies based on conserved sequence motifs (Hayden et al. 2010). Although not much is known about the ligand specificity of these subfamilies, Class I (*OR*51, *OR*52, *OR*55, and *OR*56) are largely conserved with other vertebrates (and thus hypothesized to bind to waterborne cues), and Class II (*OR*1/3/7, *OR*2/13, *OR*4, *OR*5/8/9, *OR*6, *OR*10, *OR*11, *OR*12, and *OR*14) are more mammalian specific (and thought to perhaps respond to more terrestrial cues) (Freitag et al. 1995, 1998; Hayden et al. 2010). In terms of genomic architecture of these subfamilies, they tend to be highly clustered in location and thus receptors within the same subfamily may likely experience similar evolutionary processes (Yohe et al. 2020a).

At the phenotypic level, *OR* genes in mammals are expressed in a monoallelic manner, such that a single copy of each *OR* gene is expressed per single olfactory sensory neuron (Rodriguez 2013; Monahan and Lomvardas 2015; Abdus‐Saboor et al. 2016). These neurons are embedded in olfactory epithelial tissue and distributed throughout the posterodorsal region of the nasal cavity, along with glandular supporting cells that facilitate odorant deposition (Liang 2020). Receptors bind to chemical ligands in a combinatorial fashion (Kurian et al. 2020), signal depolarization of the cell, and send converging signals to be interpreted in the olfactory bulb (Zou et al. 2009). The olfactory epithelium covers turbinal bones (turbinates), delicate, scroll‐like arrangements of approximately five bones, whose shapes can change the surface area for potential odorant deposition. Olfactory turbinals are highly convoluted and variable in shape (Ruf 2014; van Valkenburgh et al. 2014; Curtis and Simmons 2016; Lundeen and Kirk 2019), but microcomputed tomography (μCT) scanning and image analysis now makes large‐scale comparative analyses of these complex structures tractable (Yohe et al. 2018). Evidence for selection shaping the size, shape, and relative orientations of turbinates is emerging, including convergent expansion of turbinates in worm‐feeding rodents (Martinez et al. 2018) and convergent signatures of trade‐offs of olfactory and respiratory turbinates in amphibious rodents (Martinez et al. 2020). The extensive variation of olfactory turbinates may in some way be coupled with the variation within the *OR* gene family. Although ligand specificity of specific *OR*s is not well known, especially outside of model organisms, zonal expression (not turbinate specific) of particular subfamilies has been shown to respond to different volatile cues that vary in carbon atom number, as well as functional groups including aliphatic acids, aliphatic aldehydes, aliphatic alcohols, and alkanes (Mori et al. 2000). Although such a connection has never been explicitly tested, expansion of olfactory turbinates may expand OR expression, especially of specific subfamilies. The established connection of olfactory turbinates and divergent ecologies (Martinez et al. 2018, 2020) offers the opportunity to explore a relationship among evolvability of *OR* genes, olfactory morphology, and ecological constraints.

We investigate evolutionary patterns in *OR* genes and turbinates in >30 bat species (Figs. 1, S1; Tables S1 and S2) representing the ecologically diverse clade of neotropical leaf‐nosed bats (Phyllostomidae) and their close relatives within the superfamily Noctilionoidea. Noctilionoid bats show exceptional diversity in food resource consumption, occupying perhaps the widest array of dietary niches of any clade of mammals (Dumont et al. 2012). Although most echolocating bats are insectivorous, noctilionoids have diversified to specialize on arthropods, small vertebrates (e.g., fishes, frogs, birds), blood, fruit, pollen, and nectar. A suite of morphological and sensory traits is associated with divergent dietary consumption (Dumont et al. 2012; Hedrick et al. 2020; Hall et al. 2021). In concert with these changes, bats that feed on anything other than arthropods must evolve novel sensory mechanisms for finding new foods (Hall et al. 2021). Echolocation is useful for moving targets in the open air but detecting a stagnant fruit under a leaf requires a supplemental sense. Behavioral experiments of some plant‐visiting noctilionoids have demonstrated they primarily rely on olfaction and that echolocation is supplemental (Thies et al. 1998; Gonzalez‐Terrazas et al. 2016; Leiser‐Miller et al. 2020; Brokaw et al. 2021), and there is little variation in echolocation frequency among phyllostomids (Gessinger et al. 2021). Thus, in combination with numerous unique volatile compounds emitted by these plant resources (Santana et al. 2021), we expect that molecular and morphological signatures of olfactory adaptation to plant visiting should be detectable. Similar trends have been noted in insects (Dekker et al. 2006; Brand et al. 2015). For example, *Drosophila sechellia*, sister to *D. melanogaster*, specializes on ovipositing on morinda fruit and behavioral and molecular shifts in the *OR* repertoire has facilitated this specialization on a particular plant resource (Dekker et al. 2006; McBride and Arguello 2007; Auer et al. 2020). However, evidence of these olfactory adaptations and ecological shifts in vertebrates is rare, and the unusual ecological radiation of neotropical bats from a presumptive insectivorous ancestor allows us to explore these patterns in more detail.

**Figure 1:**
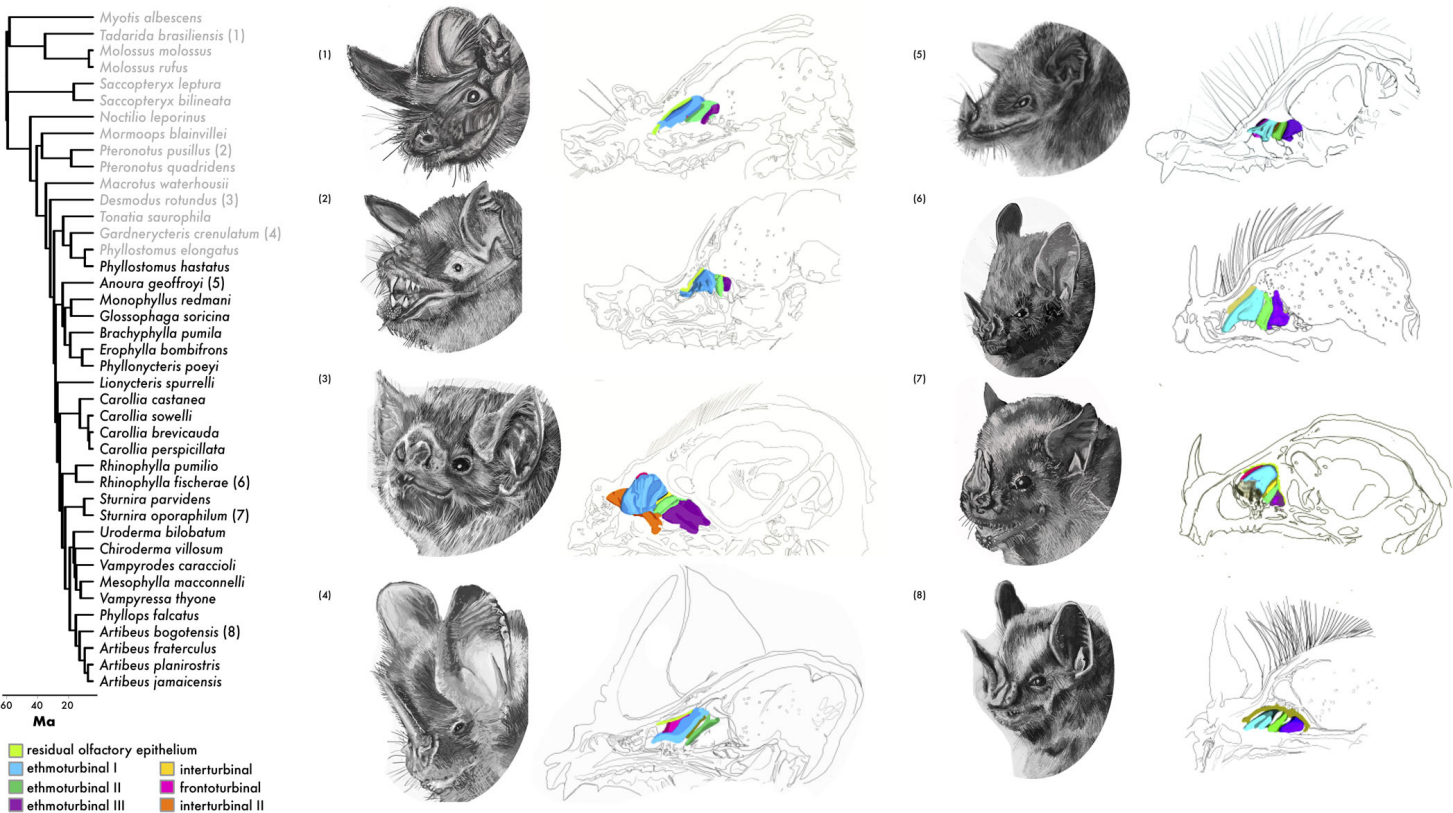
Phylogeny of cumulative taxa used in this study. Iodine‐stained μCT‐scans were used to reconstruct olfactory epithelium of different turbinates. RNA‐seq of the main olfactory epithelium was used to identify protein‐coding sequences of expressed olfactory receptors. Animal‐feeding taxa are highlighted in gray, as determined from the continuous values from Rojas et al. (2018). Numbers on the phylogeny correspond to species illustrations on the right. Illustrations on the far right are medial sagittal sections of the nasal cavity of respective species with the turbinate olfactory epithelium illustrated in separate colors. Illustrations were done by Sara Scranton.

The unstable and duplicative nature of *OR* genes as well as the highly variable features of olfactory turbinates may provide a pool of selectable variation to enable a shift into novel niches. If adaptive selection and/or novel morphologies occurred in the olfactory system prior to the evolution of consuming plant resources, then rates of evolution in *OR*s should be higher, *OR*s should have greater allelic diversity to potentially detect novel plant compounds as a result of neofunctionalization or diversifying selection after duplication, and/or divergent phenotypic optima of olfactory turbinate surface areas should be observed in plant‐visiting versus animal‐feeding bats. Alternatively, although not mutually exclusive, the extensive variation may be constrained by novel dietary niches to optimize or fine‐tune specific detection. We explore two scenarios: [1] the molecular and morphological basis of olfaction facilitated the ecological breakthrough of plant consumption (see Fig. 1a for plant‐visiting species), or [2] the constraints of finding specific plants restricted the diversity of the hypervariable olfactory system. We compared sequence variation from expressed *ORs* from olfactory epithelium transcriptomes to the surface area of olfactory epithelia from high‐resolution soft tissue μCT‐scans of over 30 species with divergent diets. This is among the first datasets of its kind, enabling us to test how ecological variation in diets might shape the evolutionary dynamics of olfactory evolvability.

## Materials and Methods

### SAMPLE COLLECTION

Specimens for both genetic and morphological analyses were collected over the course of five field expeditions: two to the Dominican Republic in 2014 and 2015 (collection permit VAPB‐01436), one to Belize in 2014 (Belize Forestry Department Scientific Research and Collecting Permit CD/60/3/14), one to Peru in 2015 (collection permit 0002287), and one to Costa Rica in 2017 (collection permit R‐041‐2017‐OT‐CONAGEBIO). All genetic tissue and morphological specimens were exported in accordance with research permit and country guidelines. Samples were imported in accordance with U.S. Center for Disease Control and U.S. Fish and Wildlife guidelines. All specimens were collected, handled, and euthanized in accordance with Stony Brook University IACUC permit 614763‐3 for Peru and 448712‐3 for Costa Rica, and Brown University IACUC 1205016 and 1504000134 and University of Georgia IACUC AUP A2009‐10003‐0 and A2014 04‐016‐Y3‐A5 for Belize.

For morphological sampling, specimens were collected on the same expeditions listed above, and many of the species replicate the samples taken for transcriptomic analyses (Table S1). Body mass was measured from living bats to serve as a proxy for body size. Individuals were euthanized and immediately placed in 4% paraformaldehyde (PFA) solution. Fixed specimens were drained and brought back to the laboratory at Stony Brook University and were immediately placed in 4% PFA again. To minimize shrinkage, we avoided putting specimens in ethanol (Hedrick et al. 2018). In 2016, specimens were placed in 10% Lugol's iodine solution (I_2_KI). Specimens were kept in solution until scanning in 2019, ranging from 2 to 3 years. A total of 30 species were sampled for morphology, and of these, 19 species had replicates for both genetic and morphological sampling.

For tissue collection for RNA‐seq, specimens were collected according to previously published protocols (Yohe et al. 2019a). Briefly, bats were euthanized using isoflurane, and cranial tissues were dissected and placed immediately in *RNAlater*. For these analyses specifically, the rostrum was clipped from the skull and the entire nasal cavity was place in *RNAlater*. The rostra were stored at 4°C overnight to ensure complete penetration of the storage solution. Samples were then placed in liquid nitrogen and transported to the lab at Stony Brook University. In the lab, rostrum samples were thawed and dissected on a cold table. Specifically, the main olfactory epithelium was removed, and a subset of this tissue was immediately used for RNA extraction. Published video dissection protocols were used to remove the olfactory epithelium (Yohe et al. 2019a; Brechbühl et al. 2011). In total, 30 species were collected for transcriptomic analyses, including one emballonurid, one molossid, two mormoopids, and 26 phyllostomids to represent a diversity of divergent diets (Figs. 1, S1; Table S2).

### μCT‐SCANNING AND TURBINATE SEGMENTATION

Formalin‐fixed museum specimens were stained in 10% Lugol's iodine solution, mounted in agarose, and scanned in the high‐resolution Nikon H225 ST μCT‐scanner. Scan parameters varied depending on specimen size and morphology, but resolution voxel size ranged from 0.01 to 0.02 mm per scan. Scan parameter details are available in Table S3. Raw μCT‐scan data were reconstructed using in‐house Nikon software to align the center of rotation and correct artifacts with beam hardening parameters. Reconstructed image stacks were imported into VGStudio version 3.3 (VGstudio Max 3.3 2014) for image segmentation of the main olfactory epithelium. When visible, the olfactory epithelium was segmented using the “magic wand” tool in the right nasal cavity on each observed turbinal and surrounding structures. Each segmented object was smoothed through “closing” each surface by a value of 1 and “eroded” by a value of −0.5. Surface areas were calculated within VGStudio after creating a region of interest of the segmented object and estimating its surface determination by setting the isovalue to completely include all segmented values (i.e., the entire histogram).

### TRANSCRIPTOMICS

RNA extraction and RNA‐seq protocols were the same as those described in a previously published study (Yohe et al. 2020a). Briefly, RNA was extracted using Qiagen RNeasy Micro Kit (ID: 74004). Samples were outsourced for cDNA library preparation and RNA sequencing. For each sample, Illumina paired‐end sequencing was performed for each cDNA library. Because tissues were collected during different field expeditions and budgets, cDNA library preparation and RNA sequencing were performed by one of several different companies, depending on which subset of samples were analyzed: University of Arizona Genetics Core Facility, BGI in China, or Novogene in China. cDNA library preparation was performed using in‐house protocols at each institution. Illumina sequencing technology improved through time, such that earlier samples resulted in read lengths of only 90 bp, whereas later samples had read lengths up to 150 bp. Depending on the company used and the timing of the sequencing, Illumina sequencing platforms varied, including HiSeq 4000, HiSeq 2500, or NovaSeq 6000. This variation likely contributes to some differences in transcript assemblies across samples. Table S4 details which sequencing platform and company was used and the read lengths obtained for each sample.

### TRANSCRIPTOME ASSEMBLY

Raw reads were trimmed, cleaned, and assembled in accordance with a previously published method (Yohe et al. 2020a). In summary, because of the duplicative nature of olfactory receptors, we implemented the Oyster River Protocol version 2.1.0 (MacManes 2018), which uses three separate assembly programs, pools assembled reads across approaches, and removes duplicate contigs. The Oyster River Protocol also provides several quantifiable measures of assembly quality, including TransRate scores (Smith‐Unna et al. 2016) that quantify coverage and segmentation of each transcript.

### OLFACTORY RECEPTOR CLASSIFICATION

The assembled transcripts for each species were run through the published program Olfactory Receptor Assigner (ORA) version 1.9.1 (Hayden et al. 2010). The ORA is a Bioperl version 1.006924 program that implements the HMMER version 3.1b (Wheeler and Eddy 2013) algorithm to characterize olfactory receptors into their respective subfamilies based on conserved binding motifs calculated by the trainer protein alignments. Although some pseudogenes were present in the transcriptomes, we limited analyses to intact genes that had the potential to be under diversifying or positive selection.

### QUANTIFYING MOLECULAR EVOLUTION

Each subfamily of *OR*s was aligned using transAlign (Bininda‐Emonds 2005) to align protein‐coding reading frames using the MAFFT version 7.388 FFT‐NS‐2 algorithm (Katoh and Standley 2013) and gap open penalty of 1.53 with an offset value of 0.123. All alignments were performed within Geneious version 10.2.3 (Kearse et al. 2012) and are available on Dryad. Best fit codon and nucleotide models were determined using ModelOMatic version 1.01 (Whelan et al. 2015) and codon and nucleotide gene trees were inferred using IQ‐TREE version 1.6.11 implementing the best fit models (Nguyen et al. 2015). Cumulative root‐to‐tip branch lengths for each tip of the codon model and nucleotide model gene trees were performed by computing the variance covariance matrix of each tree and extracting the diagonals of this matrix using ape version 5.4.1 (Paradis et al. 2004) in R.

### STATISTICAL ANALYSES OF EVOLUTIONARY RATES

Molecular evolution, specimen collections, and μCT‐scanning yielded three types of data, respectively: codon and nucleotide branch lengths, body mass, and olfactory epithelium surface area. Our goal was to integrate molecular evolutionary rates with morphological variation, but first we had to evaluate each dataset separately. We therefore implemented three sets of interrelated analyses: (1) phylogenetic regressions of the allometry between olfactory epithelium surface area and body mass, (2) regressions and principal components analyses of codon rates as a function of nucleotide rates for each gene, and (3) multivariate analyses of codon and nucleotide branch lengths together with olfactory epithelium surface area, with mass as an independent variable. For each suite of models, we also outline our predictions under either the diversifying plant‐visiting hypothesis or the evolutionary constraints hypothesis, such that once plant visiting evolved, the selection to maintain function and minimize diversity occurred in light of a highly evolvable ancestral condition.

For the first set of models, we regressed the olfactory epithelium surface area against body mass, both in the log scale to determine the evolutionary allometry of the nose anatomy. We tested a series of phylogenetic regressions with single or differing intercepts, slopes, or both depending on diet categories. We used the deviance information criterion (DIC) to assess model fit of the phylogenetic regressions. Specifically, we implemented hierarchical Bayesian models of surface area as a function of mass, both in log scale in MCMCglmm (Hadfield 2010). To account for the phylogenetic structure of errors, we included species as a group‐specific—or random—effect correlated through the inverse relatedness matrix based on the phylogenies of Shi and Rabosky (2015) and Rojas et al. (2016). To evaluate different slopes for species with different diets, we also included a group‐specific effect as a function of diet. Bat species were encoded as plant visiting if their dietary index was coded as <0, and carnivorous if >0 (Rojas et al. 2018). We implemented models with a single intercept and slope (no diet effect), separate intercepts and one slope (diet influences intercept), and with separate intercepts and slopes (diet influences intercept and slope). The latter models were implemented by estimating the variance‐covariance matrix between the intercept and the mass covariate using the us() variance structure for random effects described by Hadfield (2019), thus:

$$\begin{array}{ccc} surface.area_{j}\;∼\;mass_{j}, & & \;random\;=\;∼\;us\left(1+mass_{j}\right)\\ :group & & +species_{j},\;\text{…}, \end{array}$$

wherein surface.area is the total epithelial surface area of all turbinates for species *j*, mass is body mass (g) of the species *j*, and group is plant‐visiting/animal‐feeding (*k* = 2). The model also accounted for the phylogenetic structure of errors using a relatedness matrix based on the grafted phylogenies of Shi and Rabosky (2015) and Rojas et al. (2016). We predicted that in the diversifying scenario, the evolution of plant visiting would be coupled with an increase in olfactory surface area and extensive deviation from allometric relationships. In an evolutionary constraint scenario, we predicted that plant‐visiting bats have greater olfactory surface area, but that much of this variation is explained by body size.

For the second set of regressions, we modeled codon branch lengths as a function of nucleotide lengths. Although all models included nucleotide lengths as an independent variable, we tested for different intercepts and slopes partitioned by plant diet, multiple diet categories, gene subfamily, or species. In the first suite of models, we exclusively compared rates of molecular evolution by fitting a series of phylogenetic regressions with codon rates as a function of nucleotide rates calculated for each cumulative branch length of respective gene trees for each gene *i*. A series of models partitioning slopes (represented by group, note *k* varies depending on which group is being tested) by plant diet, multiple diet categories, gene subfamily, or species were fitted in MCMCglmm (Hadfield 2010):

$$\begin{array}{c} codon.branch.length_{i}\;∼\;nucleotide.branch.length_{i},\;random\;\\ =\;∼us\left(1+nucleotide.branch.length_{i}\right):group_{k},\text{…}. \end{array}$$

To evaluate any patterns of separation in the data not captured by the regression models, we also performed a principal components analysis of the codon and nucleotide branch lengths using the prcomp function in R. We predicted that in the diversifying scenario, plant‐visiting bats would demonstrate higher rates of codon substitutions (i.e., diversifying selection) to reflect a more diverse chemical cue profile needed to locate plant resources. In the evolutionary constraints scenario, we predicted that plant‐visiting bats may show lower rates (i.e., purifying selection) of codon substitutions, reflecting ecological constraints that need to refine the widespread high rates of molecular evolution of *OR* genes.

In the third suite of models, we related *OR* evolution and olfactory epithelium surface area by implementing multivariate models, allowing both codon branch lengths and surface area to be modeled with error. Nucleotide branch lengths and (log) body mass were both included as predictors in these phylogenetic models, with group‐specific effects outlined in the Supporting Information. In this case, the effects of the different trait responses (i.e., codon rate and surface area) were modeled as species‐specific effects that vary using the idh() variance structure, with the bat species explaining different amounts of variation for each trait, generally:

$$random\;∼\;idh\left(trait\right):species,\;rcov\;=\;∼us\left(trait\right):units\text{…}.$$

Coefficients for the multiresponse models were calculated by dividing the between‐response covariance by the trait variance, as described by Hadfield (2019). The DIC was used to select best‐fit models. Finally, all MCMCglmm models ran with and without mormoopid taxa (*n* = 3), as their skull morphology is hypervariable and may confound underlying patterns within the data (Hedrick et al. 2020). See the Supporting Information for more exploratory analyses of evolutionary rates of mormoopids versus remaining taxa. The purpose of the third suite of models was to explore if there was any relationship between genomic rates and morphological rates. If both the molecular and morphological basis of olfaction facilitated the evolution of plant consumption, then we predicted a positive correlation in plant‐visiting bats, such that plant‐visiting species would have a larger olfactory surface area and more diversifying rates of codon substitutions to reflect the need to detect a diversity of novel plant cues. Alternatively, in the constraint scenario, a negative correlation between evolutionary rates and morphological evolution may suggest stronger purifying selection in species that have evolved to rely on olfaction more heavily.

## Results

To study the variation of the olfactory system at both the morphological and molecular levels, we compared surface area of the main olfactory epithelium (*n* = 30) and used RNA‐seq (*n* = 30) of the main olfactory epithelium to sequence *OR*s in species with divergent diets, of which 18 species had both morphological and molecular data. Species were coded either as animal‐feeding (*n* = 15) or plant‐visiting (*n* = 26), based on published ecological metrics (Fig. S1; *n* = 30).

### EXCEPTIONAL VARIATION IN GROSS TURBINATE MORPHOLOGY THROUGHOUT YANGOCHIROPTERA

**Figure 2:**
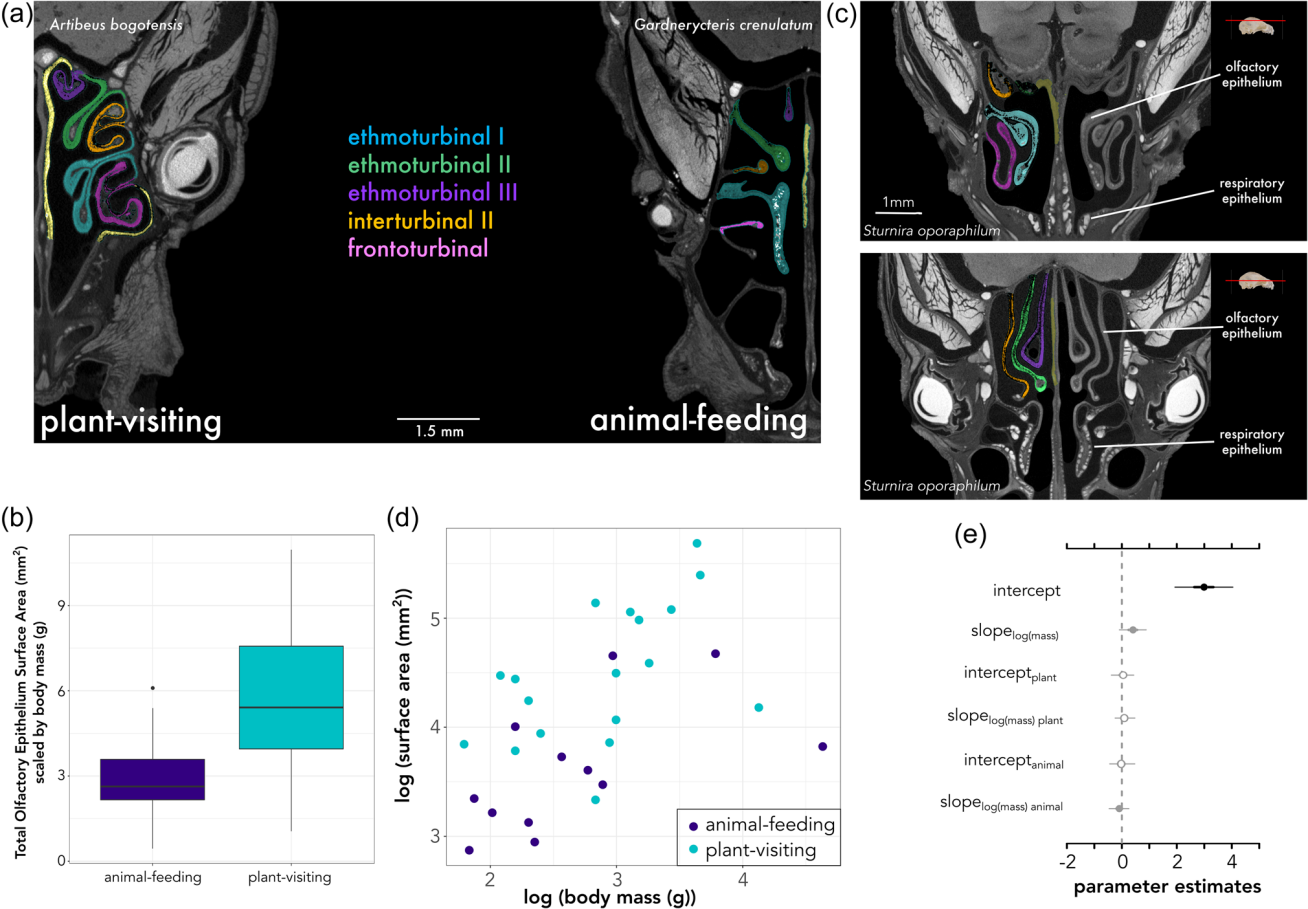
(a) Olfactory epithelium segmented from its distribution along the turbinate bones of two phyllostomid species. *Artibeus bogotensis* is an obligate frugivorous bat, whereas *Gardnerycteris crenulatum* is a specialized insectivore. (b) Comparable differences between raw data of total olfactory epithelial surface area scaled by body size among animal‐feeding and plant‐visiting bats. (c) Differences between main olfactory epithelium and respiratory epithelium observed from the iodine‐stained μCT‐scans. This example is a transverse section in *Sturnira oporaphilum*. The upper panel shows how olfactory epithelium is present on the frontoturbinal and ethmoturbinal I, but more dorsal views of the transverse section (lower panel) show that these turbinates are now covered in respiratory epithelium. Skull image from Animal Diversity Web. Colors correspond to respective turbinate bone shown in Figure 1. (d) Raw data of body mass and total olfactory epithelial surface area. (e) Parameter estimates of MCMCglmm of data from panel (b) and (d), testing for a relationship of olfactory epithelium surface area and body mass, explained by diet. Open circles denote posterior estimates overlap with zero; gray circles denote 95% credible intervals overlap with zero; and black circles indicate the entire posterior distribution is above or below zero. Note that mormoopids were removed from the analyses in panel (e).

To test whether plant‐visiting bats had more olfactory epithelia relative to animal‐feeding, we measured the surface area of the olfactory epithelium distributed in the nasal cavity from μCT‐scans of iodine‐stained specimens collected from 30 species with divergent diets (Fig. S1; Table S1). Despite extensive variation, plant‐visiting bats consistently had qualitatively more well‐developed olfactory epithelia (Fig. 2a,b), although this relationship requires control for allometry and incorporation of molecular parameters described in the following sections. Within Phyllostomidae, as well as most other previously studied members of the suborder Yangochiroptera, there are normally five turbinate bones in which the main olfactory epithelium is distributed in the nasal cavity (Bhatnagar and Kallen 1974, 1975; Yohe et al. 2018). From anterior to posterior with corresponding segmented colors (Fig. 2a), these include the frontoturbinal (pink), ethmoturbinal I (teal), interturbinal II (potentially homologous with ethmoturbinal I [pars posterior] [Ito et al. 2021]; orange), ethmoturbinal II (green), and ethmoturbinal III (purple). Residual main olfactory epithelium (yellow) can also be observed on medial parts of the nasal septum and superior portions of the nasal cavity and olfactory recess. A concern for detecting true olfactory epithelial tissue versus respiratory epithelium is warranted in bats, as the two epithelia can coexist on some turbinals. Although precise boundaries can only be determined with histology, the two can be distinguished in the diceCT scans (Fig. 2c), in which the olfactory epithelium is thick, bright, and smooth and the respiratory epithelium is more uneven with bright glandular globules are distributed throughout. Most specimens possessed the five described olfactory turbinate bones (Fig. 1), although the structures of each turbinate were highly variable. A sixth turbinal was present in two species; in *Brachyphylla pumila*, a second interturbinal (described as interturbinal I in Yohe et al. [2018]) containing dense olfactory epithelia was present between the frontoturbinal and ethmoturbinal I; and in *Desmodus rotundus*, an extra anterior turbinate bone with olfactory epithelia was observed, which we name frontoturbinal 0 to avoid confusion with the common notation of frontoturbinal for the standard most anterior turbinate bone. *Myotis albescens* and *Molossus rufus* were missing interturbinal I, but a small extra olfactory‐epithelium‐bearing turbinal was present in the posterior‐most region of the olfactory recess. This extra turbinal was not present in the congeneric *Molossus molossus*.

### ROBUST EVIDENCE FOR ALLOMETRY, WEAK EVIDENCE OF SELECTION IN OLFACTORY EPITHELIUM SURFACE AREA

We first explored how olfactory epithelial surface area related to plant visiting in bats, with the prediction that plant‐visiting bats would have increased surface area relative to animal‐feeding, independent of body size, which would support a diversifying evolutionary scenario. Several comparative methods to quantify this relationship were explored and are discussed in more detail in the Supporting Information. We used body mass (g) measured directly from the live specimen in the field as the proxy for size (Fig. 2d). When testing whether olfactory epithelial surface area was different in plant‐ and animal‐feeding bats, as well as controlling for body size and exploring how it may relate to diversity in olfactory epithelium surface area, analyses of allometric scaling using phylogenetic regressions found a model with different intercepts and slopes by plant feeding to best fit the data (Table S5; DIC = 52.6 vs. DIC > 53 for simpler models [i.e., single slope/intercept]), suggesting differences between the two groups. Without the mormoopids (Fig. 2e), posterior estimates of the allometric slope overlapped with those obtained using directional models (mean slope = 0.39, lower = 0.04, upper = 0.75). There was a trend toward higher slopes for plant‐visiting species (mean slope = 0.094, lower = 0.089, upper = 0.47) compared to animal‐feeding ones (mean slope = −0.098, lower = −0.46, upper = −0.93; Fig. 2c). Given that body mass explained much of the relationship, differences among plant‐visiting and animal‐feeding were small, supporting the constraint hypothesis. Including all taxa, results were similar, except posterior estimates of the allometric slope that were higher (mean slope = 0.47, lower = 0.11, upper = 0.93; Fig. S2).

### OR CODON EVOLUTION EXPLAINED BY OR SUBFAMILY AND NUCLEOTIDE SUBSTITUTIONS, NOT ECOLOGY

Plant‐visiting bats may require a diverse or faster evolving repertoire of olfactory receptors because they rely on complex plant volatile bouquets for their food detection. We tested this hypothesis by sequencing the transcriptomes of the main olfactory epithelium, identifying intact olfactory receptor genes, and comparing among plant and animal feeders. High‐coverage RNA‐seq data (Fig. S1; Tables S4 and S6) were obtained and intact olfactory receptors were identified and classified according to their respective subfamilies. Of the 30 species, an average of 221 (±95) *OR*s were detected, with large variation among species (Fig. S1; Table S7). *Mormoops blainvillei* had only one intact reading frame and, potentially due to low detection, was removed from downstream analyses. Intriguingly, only intact trace‐amine‐associated receptors, another gene family of chemoreceptors, were detected in *M. blainvillei*. We report this here and deeper investigation is warranted, although this is outside the scope of this analysis. There was a weak positive relationship (slope = 0.004 ± 0.002; *F*_1, 28)_ = 4.6; *P* = 0.041) between number of *OR*s detected and RNA Integrity Number (Fig. S3). Because a previous study found that transcriptomes of the main olfactory epithelium only recover 50%–60% of total intact *OR* genes (Yohe et al. 2020a), and in addition to high rates of duplication and low rates of homology among *OR*s, incomplete RNA‐seq data may confound comparisons of numbers of receptors across species. Instead, we measured rates of evolution for each gene per species. Alignment and best‐fit substitution models of evolution are available in Table S8.

**Figure 3:**
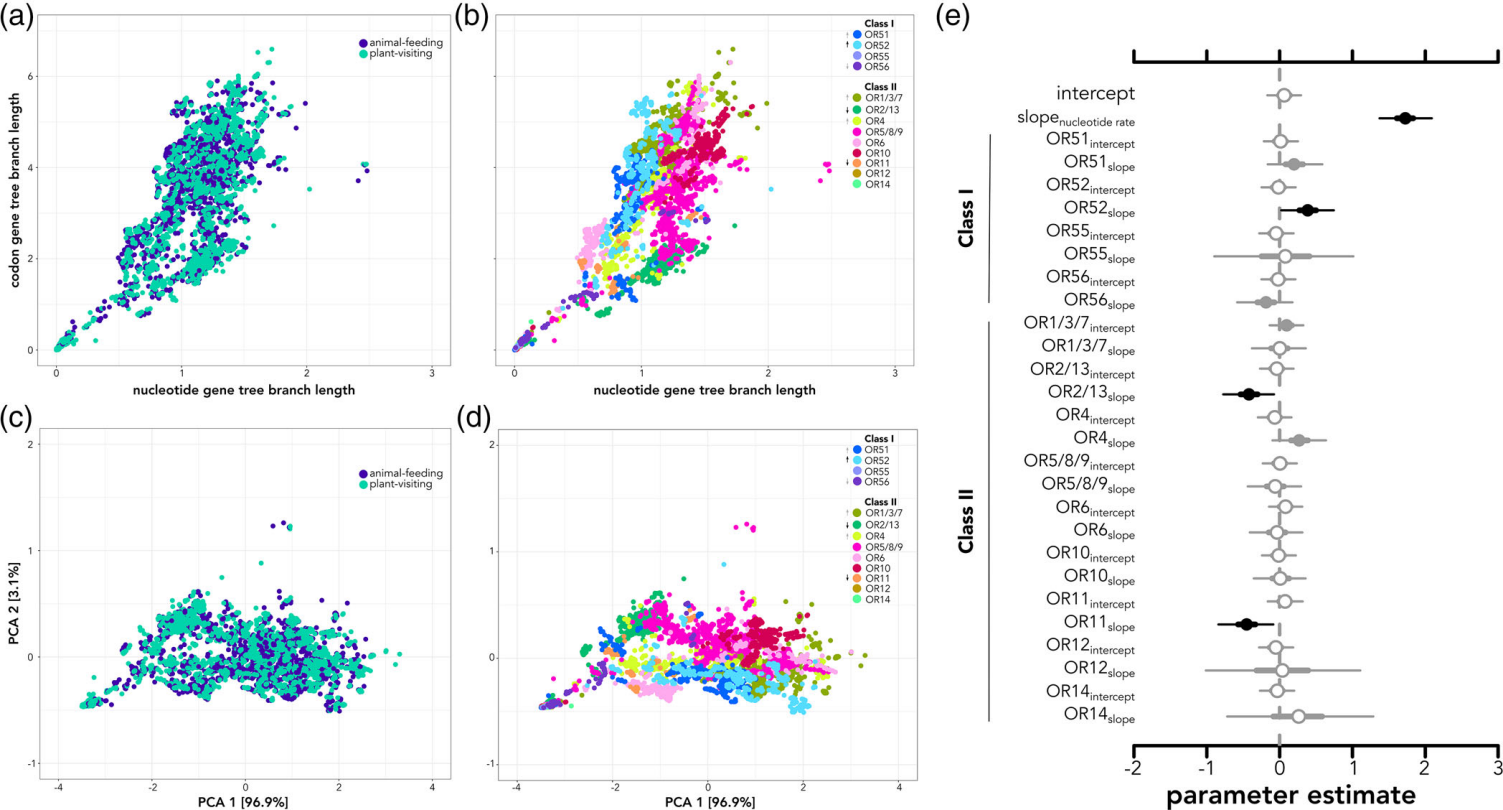
Branch length estimates of each *olfactory receptor* (*OR*) gene plotted as nucleotide rates versus codon model rates and colored by (a) diet and (b) *OR* subfamily. PCA axes of codon and nucleotide branch lengths colored by (c) diet and (d) *OR* subfamily. Posterior distribution parameter estimates (e) of hierarchical models testing for relationship of *OR* subfamily and nucleotide branch lengths with codon branch length. Open circles denote posterior estimates overlap with zero; gray circles denote 95% credible intervals overlap with zero; and black circles indicate the entire posterior distribution is above or below zero. Arrows in panels (b) and (d) correspond to higher or lower rates of evolution as shown in panel (e).

To measure differences in rates of evolution between animal‐feeding and plant‐visiting bats, we used cumulative root‐to‐tip branch lengths for several reasons. First, comparing codon and nucleotide rates from their corresponding trees is conceptually similar to measures of molecular selection such as ratios of rates of nonsynonymous substitutions (*dN*) to rates of synonymous substitution (*dS*) (Yohe et al. 2020b). Second, this method has the added advantage of incorporating both codon and different nucleotide substitution parameters into the best‐fit models, adding parameters such as transition and transversion rate ratios when appropriate to the dataset. In this case, codon models were used instead of amino acid substitution models, as the former were better fits for all olfactory receptor subfamilies. Third, and crucially, the branch length approach helps overcome the issue of determining true orthology versus paralogy, which is very challenging in large gene families. Resulting branch lengths in nucleotide substitutions per codon site for codon‐based trees and nucleotide substitutions per site for nucleotide trees are directly comparable across the entire phylogeny. The best‐fit model of codon lengths as a function of nucleotide lengths including mormoopids partitioned both intercepts and slopes by gene subfamily (Table S9; DIC = −18,085; Fig. 3). There was no support for partitioning intercepts or slopes by plant diet, diet categories, or species (Table S9; DIC > −11,433; Fig. 3a). This means that there was no meaningful difference between diet groups and that *OR* subfamily explained the rates of evolution, rejecting our prediction that diversifying selection (increased codon substitutions) would be coupled with the evolution of plant visiting. With the best‐fit model, we detected a higher slope in the codon rate for *OR* subfamily 52, and lower slopes for subfamilies 11 and 2/13 (Fig. 3b,e). The resulting model captured important differences in rate scaling across gene subfamilies, as shown in comparisons between observed and predicted values (Fig. S4). The PCA found 96.1% of the variation was loaded in the first principal component, with most of the variation explained by the codon branch lengths. When visualizing clusters within the PCA axes, there was no clustering by diet (Fig. 3c) but clear clustering of different *OR* subfamilies (Fig. 3d).

### INVERSE RELATIONSHIP BETWEEN OR EVOLUTION AND OLFACTORY EPITHELIUM SURFACE AREA

**Figure 4:**
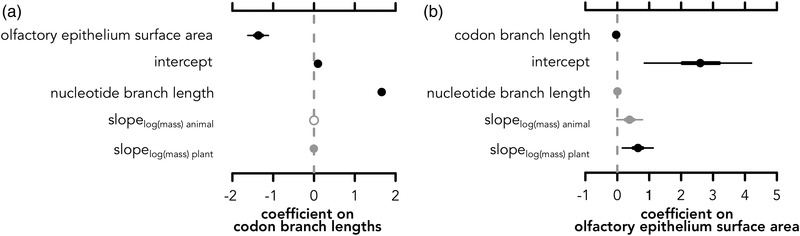
Posterior distributions of parameter estimates of hierarchical models from analyses combining molecular and morphological data. (a) Estimated coefficients on codon branch lengths and (b) estimated coefficients of covariates on olfactory epithelium surface area. Open circles denote posterior estimates overlap with zero; gray circles denote 95% credible intervals overlap with zero; and black circles indicate the entire posterior distribution is above or below zero. To interpret these plots, when a coefficient posterior is above zero, there is a positive relationship with the response, and when it is below zero, there is a negative relationship with the response. Note that although the codon branch length coefficient appears small, this is due to the units of change of codon branch lengths being much smaller than units of change of surface area. What is important is that the coefficient of codon branch lengths is positive, including the entire posterior distribution; it is challenging to visualize with the intercept being so large as well.

Finally, we tested whether there was a molecular‐morphological relationship that may explain differences in diet. In multiresponse models, both codon branch lengths and olfactory epithelium surface area are responses with their own modeled errors. Thus, the estimated coefficients must be interpreted in a multivariate framework. The best multiresponse model including mormoopids (Table S10; DIC: −37343) only had a weak trend for log body mass of plant‐eating bats relating to codon rates (mean slope = −0.0038, lower = −0.0128, upper = 0.0042, Figs. 4, S5, including mormoopids). When excluding mormoopids, the best multiresponse model (Table S10; DIC = −35451) found a strong inverse relationship between codon rates (mean slope: −0.034, lower = −0.042, upper = −0.028; Fig. 4a) and olfactory epithelium surface area (slope: −1.36, lower = −1.62, upper = −1.12; Fig. 4b), supporting the constraint hypothesis. After accounting for phylogeny, codon tree branch lengths, and body mass, the coefficients of body mass on olfactory epithelium surface area for both animal‐feeding (mean = 0.38, lower = −0.012, upper = 0.79) and plant‐visiting bats are positive, but substantially higher for plant‐visiting bats (Fig. 4b; mean = 0.68, lower = 0.23, upper 0.65). Both coefficients being positive suggest that body mass has a positive relationship on olfactory epithelial surface area (see Fig. 3d), and differences between animal feeding and plant visiting are only present when accounting for both body size and codon branch lengths.

## Discussion

Highly evolvable genes and phenotypes are often associated with exploratory systems, for which variation does not come at the same potential fitness cost as they do for central core processes (Kirschner and Gerhart 1998). Yet, when novel variable mutants are favored in a given niche, environmental conditions may subsequently constrain that variation derived from mutation and purifying selection now displaces previously neutral processes (Kirschner and Gerhart 1998). Although previous emphasis has been on the unstable genomic architecture (i.e., arrangement of functional elements, highly duplicated segments, etc. [Koonin 2009]) underlying highly evolvable genes and traits, the operation of environmental constraints on this variation is less understood. Using the highly evolvable olfactory system in a clade of bats with divergent dietary ecologies, we have discovered that, although there is exceptional variation in both olfactory morphology (Fig. 2) and *OR* genes (Fig. 3), bats that use plant resources show an inverse relationship between rates of molecular and morphological evolution (Fig. 4). Having hypothesized a single expansion or shift to facilitate plant visiting, we expected strong association of molecular rates and morphological differences with plant visiting (i.e., Fig. 2d would show clear differences in plant‐visiting bats independent of body size; Figure 3a,c would have revealed differences in rates of molecular evolution between plant feeders and animal feeders). Instead, we found shorter *OR* molecular branch lengths in bats with larger epithelial surface area (Fig. 4), despite ubiquitous elevated rates of molecular and morphological evolution (Figs. 2, 3). We propose that once bats evolved plant‐visiting, the exploratory background of a rapidly evolving olfactory system was suddenly exposed to strong selection for maintenance of the ability to detect specific plant odorants. This “slowdown” could be important for fine‐tuning associations with plants to optimize for detecting fruit ripeness, floral blooms, and/or avoiding toxicity.

Without considering morphology, a strong association between evolutionary codon‐to‐nucleotide rate with *OR* subfamily (Fig. 3b,d,e) suggests most of the variation in *OR*s is endogenous and explained by intrinsic genomic processes, instead of ecology (Fig. 3a,c); some subfamilies (e.g., OR52, OR4) are evolving at faster rates than others. Within genomes, loci within *OR* subfamilies tend to be highly clustered, and in bats, many times the entire *OR* subfamily was detected within a single scaffold (Yohe et al. 2020a). This highly clustered nature is caused by rampant tandem duplication (Niimura and Nei 2005), which contributes to the unstable genomic architecture of the system. We hypothesize this instability is the genetic mechanism that generates exceptional variation in chemosensory genes, and that *OR* genes (and likely other chemosensory receptor genes) are not as constrained as most protein‐coding genes (Arguello et al. 2016). Most OR proteins are highly specific and are not involved in core cellular pathways (i.e., they have minimal pleiotropy) (Arguello et al. 2016). Their main function is to initiate G‐protein‐coupled receptor pathway responses and to “survey” and respond to environmental chemical cues (i.e., they are, as pathogen‐detection, exploratory proteins). Thus, we predict that duplication of *OR* genes does not have strong dosage effects. Instead, duplication might increase the probability of expression for a given receptor or increase the genomic substrate for new mutations to arise. Indeed, it is the standing variation within these contingency loci that contributes to the “adaptability” of chemosensory receptor genes in divergent *Drosophila* populations (Arguello et al. 2016).

The genetic controls of olfactory turbinate morphogenesis are unrelated to *OR* genes (although related the olfactory bulb) (Treloar et al. 2010), but the expansion of olfactory epithelium surface area directly increases the neural epithelial space in which olfactory receptor neurons can express *OR* genes. Although the expression of *OR* genes is monoallelic and stochastic per sensory neuron (Rodriguez 2013; Monahan and Lomvardas 2015; Abdus‐Saboor et al. 2016), there is zonal organization of expression within the turbinates associated with different *OR* subfamilies. This zonation is complex in three‐dimensional space. *OR* gene subfamilies are not distributed on specific turbinates, but instead spatially distributed across turbinates in space (Coleman et al. 2019). The more outward parts of the turbinates express similar receptor families compared to zones closer to the olfactory bulb (Mori et al. 2000). Although further research both establishing the boundaries of these zones and the functional differences among *OR* subfamilies regarding odorant molecule binding is necessary to properly interpret differences in relation to evolutionary niche divergence, our study identifies a key relationship between morphology and *OR* gene repertoire. For example, given our inverse relationship of codon rates and surface area, the increased elevated rates in *OR*52 may be coupled with a decrease in the zonal region of Class II expressing genes and the more conserved signatures of *OR*2/13 may relate to increased zonal expression of Class II epithelia across turbinates. Indeed, *OR*2/13 has been implicated as a significant outlier in frugivorous phyllostomids (Hayden et al. 2014), albeit this result was based on receptor counts from a method known to significantly underestimate gene duplicate counts (Yohe et al. 2020a). Further zonal mapping of receptor subfamilies in bats will be necessary to confirm these hypotheses, but we encourage future studies investigating these reported trends. Modeling errors in both morphology and genes simultaneously (although also accounting for allometry and phylogeny) in a Bayesian hierarchical framework revealed strong and inverse relationships between protein‐coding evolutionary rates and surface area among both plant‐visiting and animal‐feeding bats, with a stronger body mass allometry in the former (Fig. 4). This corroborates our hypothesis that chemosensory system evolution is confounded by high variation that must be accounted for when deciphering evolutionary patterns.

It has been previously hypothesized that olfactory key innovations enabled (and continue to enable) the detection of new plant compounds (Hayden et al. 2014). Based on our results, we now hypothesize that standing variation in highly evolvable *OR* genes and morphology is fine‐tuned in plant‐visiting phyllostomid bats. Indeed, a recent analysis recently noted a refinement of *OR* diversity at shallower evolutionary scales within a sympatric genus (Yohe et al. 2021). A complex interplay of hypervariable morphology (Fig. 2) and receptor repertoire (Fig. 3) may have been ideal for exploring novel niches. However, once shifts into more specialized adaptive zones occurred, selection prevented further extensive change of *OR*s perhaps to maintain a repertoire that can recognize a diverse but consistent mix of odorant cues. Expanded olfactory epithelial surface area may enable more expression of these conserved, more slowly evolving receptors (Fig. 4).

Within the phyllostomid radiation and its close relatives, patterns beyond olfaction support this hypothesis. For morphology, the shift from an insectivorous ancestor to a derived plant specialist is supported by transitional fossils (i.e., omnivorous ancestors) (Yohe et al. 2015), even early within the superfamily radiation (e.g., †*Vulcanops jennyworthyae*, an omnivorous burrower [Hand et al. 2018]). Most craniofacial variation occurs late in development, suggesting the palate and nasal cavity regions have fewer constraints and could facilitate morphological evolvability (Camacho et al. 2019). Major transitions in sensory traits occurred early in the radiation, whereas mechanical feeding shifts were more recent (Hall et al. 2021). At the molecular level, positive selection in vision and diet‐related genes occurred mostly at the origins of Phyllostomidae and their relatives, instead of at nodes of dietary shifts toward plant visiting (Davies et al. 2020; Potter et al. 2021). Thus, a “backbone” of extensive variation linked to omnivory may have set the stage for later shifts to highly specialized diets. In either case, an inverse relationship between morphology and protein‐coding evolutionary rates emerged only after controlling for extensive sources of intrinsic variation within the system. This intriguing pattern warrants further investigation of the interplay among *OR* expression, the distribution of the tissue expressing these genes, and how evolution shapes both and their interaction.

## AUTHOR CONTRIBUTIONS

LRY designed the study, collected the field samples, generated morphological and molecular data, analyzed the data, and wrote the manuscript. MF and DL performed critical morphological data collection and edited the manuscript. KTJ contributed data analyses and edited the manuscript. TPY wrote and implemented scripts critical to data analyses. MKRS, EMR, and NBS collected samples and edited the manuscript. RH, GM, BPH, AS, KES, and ED provided guidance in data analysis and interpretation and edited the manuscript. SJR assisted in field sample collection, data interpretation, and edited the manuscript. BASB provided resource infrastructure and analytical guidance and edited the manuscript. LMD assisted in field collection, statistical analyses, and wrote the manuscript.

## CONFLICT OF INTEREST

The authors declare no conflict of interest.

## DATA ARCHIVING

Alignments have been deposited to Dryad (https://doi.org/10.5061/dryad.bvq83bkc8). Raw reads from the transcriptome data have been deposited into the NCBI Sequence Read Archive and μCT‐scans have been deposited to Morphosource. Links to the supplemental material archives are available in the Supporting Information.

Associate Editor: Dr. C. Linnen

Handling Editor: Dr. M. Zelditch

## Supporting information

Supplementary Information

## References

[evo14591-bib-0001] Abdus‐Saboor, I. , Al Nufal, M.J. , Agha, M.V. , Ruinart De Brimont, M. , Fleischmann, A. & Shykind, B.M. (2016) An expression refinement process ensures singular odorant receptor gene choice. Current Biology, 26, 1083–1090. 27040780 10.1016/j.cub.2016.02.039 PMC12952689

[evo14591-bib-0002] Arguello, J.R. , Cardoso‐Moreira, M. , Grenier, J.K. , Gottipati, S. , Clark, A.G. & Benton, R. (2016) Extensive local adaptation within the chemosensory system following *Drosophila melanogaster*’s global expansion. Nature Communications, 7, ncomms11855. 10.1038/ncomms11855 PMC4910016 27292132

[evo14591-bib-0003] Auer, T.O. , Khallaf, M.A. , Silbering, A.F. , Zappia, G. , Ellis, K. , Álvarez‐Ocaña, R. , Arguello, J.R. , Hansson, B.S. , Jefferis, G.S.X.E. , Caron, S.J.C. , et al. (2020) Olfactory receptor and circuit evolution promote host specialization. Nature, 579, 402–408. 32132713 10.1038/s41586-020-2073-7 PMC7100913

[evo14591-bib-0004] Bhatnagar, K.P. & Kallen, F.C. (1974) Morphology of the nasal cavities and associated structures in *Artibeus jamaicensis* and *Myotis lucifugus* . The American Journal of Anatomy, 139, 167–189. 4812217 10.1002/aja.1001390203

[evo14591-bib-0005] Bhatnagar, K.P. & Kallen, F.C. (1975) Quantitative observations on the nasal epithelia and olfactory innervation in bats. Suggested design mechanisms for the olfactory bulb. Acta Anatomica, 91(2), 272–282. 1146482 10.1159/000144389

[evo14591-bib-0006] Bininda‐Emonds, O.R.P. (2005) transAlign: using amino acids to facilitate the multiple alignment of protein‐coding DNA sequences. BMC Bioinformatics, 6, 1–6. 15969769 10.1186/1471-2105-6-156 PMC1175081

[evo14591-bib-0007] Brand, P. , Ramírez, S.R. , Leese, F. , Quezada‐Euan, J.J.G. , Tollrian, R. & Eltz, T. (2015) Rapid evolution of chemosensory receptor genes in a pair of sibling species of orchid bees (Apidae: Euglossini). BMC Evolutionary Biology, 15, 176. 26314297 10.1186/s12862-015-0451-9 PMC4552289

[evo14591-bib-0008] Brechbühl, J. , Luyet, G. , Moine, F. , Rodriguez, I. & Broillet, M.‐C. (2011) Imaging pheromone sensing in a mouse vomeronasal acute tissue slice preparation. J. Vis. Exp, 58, e3311. 10.3791/3311 PMC3369656 22157638

[evo14591-bib-0009] Brokaw, A.F. , Davis, E. , Page, R.A. & Smotherman, M. (2021) Flying bats use serial sampling to locate odour sources. Biology Letters 17. 10.1098/rsbl.2021.0430 PMC8526173 34665992

[evo14591-bib-0010] Camacho, J. , Heyde, A. , Bhullar, B.A.S. , Haelewaters, D. , Simmons, N.B. & Abzhanov, A. (2019) Peramorphosis, an evolutionary developmental mechanism in neotropical bat skull diversity. Developmental Dynamics, 248, 1129–1143. 31348570 10.1002/dvdy.90

[evo14591-bib-0011] Coleman, J.H. , Lin, B. , Louie, J.D. , Peterson, J. , Lane, R.P. & Schwob, J.E. (2019) Spatial determination of neuronal diversification in the olfactory epithelium. The Journal of Neuroscience, 39, 814–832. 30530861 10.1523/JNEUROSCI.3594-17.2018 PMC6382982

[evo14591-bib-0012] Curtis, A.A. & Simmons, N.B. (2016) Unique turbinal morphology in horseshoe bats (Chiroptera: Rhinolophidae). The Anatomical Record, 300(2), 309–325. 27863117 10.1002/ar.23516

[evo14591-bib-0013] Davies, K.T.J. , Yohe, L.R. , Almonte, J. , Sánchez, M.K.R. , Rengifo, E.M. , Dumont, E.R. , Sears, K.E. , Dávalos, L.M. & Rossiter, S.J. (2020) Foraging shifts and visual preadaptation in ecologically diverse bats. Molecular Ecology, 29, 1839–1859. 32293071 10.1111/mec.15445

[evo14591-bib-0014] Dekker, T. , Ibba, I. , Siju, K.P. , Stensmyr, M.C. & Hansson, B.S. (2006) Olfactory shifts parallel superspecialism for toxic fruit in *Drosophila melanogaster* sibling, *D. sechellia* . Current Biology, 16, 101–109. 16401429 10.1016/j.cub.2005.11.075

[evo14591-bib-0015] Doty, R.L. (1986) Odour‐guided behaviour in mammals. Experientia, 42, 257–271. 3514263 10.1007/BF01942506

[evo14591-bib-0016] Dumont, E.R. , Davalos, L.M. , Goldberg, A. , Santana, S.E. , Rex, K. & Voigt, C.C. (2012) Morphological innovation, diversification and invasion of a new adaptive zone. The American Journal of Anatomy, 279, 1797–1805. 10.1098/rspb.2011.2005 PMC3297451 22113035

[evo14591-bib-0017] Feiner, N. , Brun‐Usan, M. & Uller, T. (2021) Evolvability and evolutionary rescue. Evolution & Development 1–12. 10.1111/ede.12374 33528902

[evo14591-bib-0018] Freitag, J. , Krieger, J. , Strotmann, J. & Breer, H. (1995) Two classes of olfactory receptors in *Xenopus laevis* . Neuron, 15, 1383–1392. 8845161 10.1016/0896-6273(95)90016-0

[evo14591-bib-0019] Freitag, J. , Ludwig, G. , Andreini, I. , Rössler, P. & Breer, H. (1998) Olfactory receptors in aquatic and terrestrial vertebrates. Journal of Comparative Physiology, 183, 635–650. 9839455 10.1007/s003590050287

[evo14591-bib-0020] Gessinger, G. , Page, R. , Wilfert, L. , Surlykke, A. , Brinkløv, S. & Tschapka, M. (2021) Phylogenetic patterns in mouth posture and echolocation emission behavior of phyllostomid bats. Frontiers in Ecology and Evolution, 9, 1–15.

[evo14591-bib-0021] Gonzalez‐Terrazas, T.P. , Martel, C. , Milet‐Pinheiro, P. , Ayasse, M. , Kalko, E.K.V. & Tschapka, M. (2016) Finding flowers in the dark: nectar‐feeding bats integrate olfaction and echolocation while foraging for nectar. Royal Society Open Science, 3. 10.1098/rsos.160199 PMC5108945 27853595

[evo14591-bib-0022] Graves, C.J. , Ros, V.I.D. , Stevenson, B. , Sniegowski, P.D. & Brisson, D. (2013) Natural selection promotes antigenic evolvability. PLoS Pathogens, 9, e1003766. 24244173 10.1371/journal.ppat.1003766 PMC3828179

[evo14591-bib-0023] Hadfield, J.D. (2019) MCMCglmm course notes. Unpublished Manuscript, University of Edinburgh, Edinburgh, U.K.

[evo14591-bib-0024] Hadfield, J.D. (2010) MCMC methods for multi‐response generalized linear mixed models: the MCMCglmm R package. J. Stat. Softw, 33, 1–22.20808728

[evo14591-bib-0025] Hall, R.P. , Mutumi, G.L. , Hedrick, B.P. , Yohe, L.R. , Sadier, A. , Davies, K.T.J. , Rossiter, S.J. , Sears, K. , Dávalos, L.M. & Dumont, E.R. (2021) Find the food first: an omnivorous sensory morphotype predates biomechanical specialization for plant based diets in phyllostomid bats. Evolution (N. Y), 75, 2791–2801. 10.1111/evo.14270 34021589

[evo14591-bib-0026] Hand, S.J. , Beck, R.M.D. , Archer, M. , Simmons, N.B. , Gunnell, G.F. , Scofield, R.P. , Tennyson, A.J.D. , De Pietri, V.L. , Salisbury, S.W. & Worthy, T.H. (2018) A new, large‐bodied omnivorous bat (Noctilionoidea: Mystacinidae) reveals lost morphological and ecological diversity since the Miocene in New Zealand. Scientific Reports, 8, 235. Springer US. 29321543 10.1038/s41598-017-18403-w PMC5762892

[evo14591-bib-0027] Hayden, S. , Bekaert, M. , Crider, T.A. , Mariani, S. , Murphy, W.J. & Teeling, E.C. (2010) Ecological adaptation determines functional mammalian olfactory subgenomes. Genome Research, 20, 1–9. 19952139 10.1101/gr.099416.109 PMC2798820

[evo14591-bib-0028] Hayden, S. , Bekaert, M. , Goodbla, A. , Murphy, W.J. , Dávalos, L.M. & Teeling, E.C. (2014) A cluster of olfactory receptor genes linked to frugivory in bats. Molecular Biology and Evolution, 31, 917–927. 24441035 10.1093/molbev/msu043

[evo14591-bib-0029] Hedrick, B.P. , Yohe, L. , Vander Linden, A. , Dávalos, L.M. , Sears, K. , Sadier, A. , Rossiter, S.J. , Davies, K.T.J. & Dumont, E. (2018) Assessing soft‐tissue shrinkage estimates in museum specimens imaged with diffusible iodine‐based contrast‐enhanced computed tomography (diceCT). Microscopy and Microanalysis, 24, 284–291. 29916341 10.1017/S1431927618000399

[evo14591-bib-0030] Hedrick, B.P. , Mutumi, G.L. , Munteanu, V.D. , Sadier, A. , Davies, K.T.J. , Rossiter, S.J. , Sears, K.E. , Dávalos, L.M. & Dumont, E. (2020) Morphological diversification under high integration in a hyper diverse mammal clade. Journal of Mammalian Evolution, 27, 563–575.

[evo14591-bib-0031] Ito, K. , Tu, V.T. , Eiting, T.P. , Nojiri, T. & Koyabu, D. (2021) On the embryonic development of the nasal turbinals and their homology in bats. Frontiers in Cell and Developmental Biology, 9, 1–19. 10.3389/fcell.2021.613545 PMC8021794 33834019

[evo14591-bib-0032] Katoh, K. & Standley, D.M. (2013) MAFFT multiple sequence alignment software version 7: improvements in performance and usability. Molecular Biology and Evolution, 30, 772–780. 23329690 10.1093/molbev/mst010 PMC3603318

[evo14591-bib-0033] Kearse, M. , Moir, R. , Wilson, A. , Stones‐Havas, S. , Cheung, M. , Sturrock, S. , Buxton, S. , Cooper, A. , Markowitz, S. , Duran, C. , et al. (2012) Geneious Basic: an integrated and extendable desktop software platform for the organization and analysis of sequence data. Bioinformatics (Oxford, England), 28, 1647–1649. 22543367 10.1093/bioinformatics/bts199 PMC3371832

[evo14591-bib-0034] Kirschner, M. & Gerhart, J. (1998) Evolvability. Proceedings of the National Academy of Sciences U.S.A, 95, 8420–8427. 10.1073/pnas.95.15.8420 PMC33871 9671692

[evo14591-bib-0035] Koonin, E.V. (2009) Evolution of genome architecture. The International Journal of Biochemistry & Cell Biology, 41, 298–306. 18929678 10.1016/j.biocel.2008.09.015 PMC3272702

[evo14591-bib-0036] Kurian, S.M. , Naressi, R.G. , Manoel, D. , Barwich, A.S. , Malnic, B. & Saraiva, L.R. (2020) Odor coding in the mammalian olfactory epithelium. Cell and Tissue Research, 383(1), 445–456. 10.1007/s00441-020-03327-1 PMC7873010 33409650

[evo14591-bib-0037] Kurian, S.M. , Naressi, R.G. , Manoel, D. , Barwich, A.S. , Malnic, B. & Saraiva, L.R. (2021) Odor coding in the mammalian olfactory epithelium. Cell and Tissue Research, 383(1), 445–456. 33409650 10.1007/s00441-020-03327-1 PMC7873010

[evo14591-bib-0038] Leiser‐Miller, L. , Kaliszewska, Z. , Lauterbur, M. , Mann, B. , Riffell, J. & Santana, S.E. (2020) A fruitful endeavor: scent cues and echolocation behavior used by *Carollia castanea* to find fruit. Integrative Organismal Biology, 2. 10.1093/iob/obaa007 PMC7671165 33791551

[evo14591-bib-0039] Liang, F. (2020) Sustentacular cell enwrapment of olfactory receptor neuronal dendrites: an update. Genes, 11, 14–17. 10.3390/genes11050493 PMC7291085 32365880

[evo14591-bib-0040] Lundeen, I.K. & Kirk, E.C. (2019) Internal nasal morphology of the Eocene primate *Rooneyia viejaensis* and extant Euarchonta: using μCT scan data to understand and infer patterns of nasal fossa evolution in primates. Journal of Human Evolution, 132, 137–173. 31203844 10.1016/j.jhevol.2019.04.009

[evo14591-bib-0041] MacManes, M.D. (2018) The Oyster River Protocol: a multi‐assembler and kmer approach for *de novo* transcriptome assembly. PeerJ, 6, e5428. 30083482 10.7717/peerj.5428 PMC6078068

[evo14591-bib-0042] Martinez, Q. , Lebrun, R. , Achmadi, A.S. , Esselstyn, J.A. , Evans, A.R. , Heaney, L.R. , Miguez, R.P. , Rowe, K.C. & Fabre, P.H. (2018) Convergent evolution of an extreme dietary specialisation, the olfactory system of worm‐eating rodents. Scientific Reports, 8, 1–13. 30546026 10.1038/s41598-018-35827-0 PMC6293001

[evo14591-bib-0043] Martinez, Q. , Clavel, J. , Esselstyn, J.A. , Achmadi, A.S. , Grohé, C. , Pirot, N. & Fabre, P.‐H. (2020) Convergent evolution of olfactory and thermoregulatory capacities in small amphibious mammals. Proceedings of the National Academy of Sciences U.S.A. 117. 10.1073/pnas.1917836117 PMC7183214 32253313

[evo14591-bib-0044] McBride, C.S. & Arguello, J.R. (2007) Five *Drosophila* genomes reveal nonneutral evolution and the signature of host specialization in the chemoreceptor superfamily. Genetics, 177, 1395–1416. 18039874 10.1534/genetics.107.078683 PMC2147975

[evo14591-bib-0045] Merrikh, C.N. & Merrikh, H. (2018) Gene inversion potentiates bacterial evolvability and virulence. Nature Communications, 9(1), 1–10. 10.1038/s41467-018-07110-3 PMC6220195 30405125

[evo14591-bib-0046] Monahan, K. & Lomvardas, S. (2015) Monoallelic expression of olfactory receptors. Annual Review of Cell and Developmental Biology, 31, 721–740. 10.1146/annurev-cellbio-100814-125308 PMC4882762 26359778

[evo14591-bib-0047] Mori, K. , Von Campenhausen, H. & Yoshihara, Y. (2000) Zonal organization of the mammalian main and accessory olfactory systems. Philosophical Transactions of the Royal Society B: Biological Sciences, 355, 1801–1812. 10.1098/rstb.2000.0736 PMC1692907 11205342

[evo14591-bib-0048] Nei, M. & Rooney, A.P. (2005) Concerted and birth‐and‐death evolution of multigene families. Annual Review of Genetics, 39, 121–152. 10.1146/annurev.genet.39.073003.112240 PMC1464479 16285855

[evo14591-bib-0049] Nguyen, L.T. , Schmidt, H.A. , Von Haeseler, A. & Minh, B.Q. (2015) IQ‐TREE: a fast and effective stochastic algorithm for estimating maximum‐likelihood phylogenies. Molecular Biology and Evolution, 32, 268–274. 25371430 10.1093/molbev/msu300 PMC4271533

[evo14591-bib-0050] Niimura, Y. (2012) Olfactory receptor multigene family in vertebrates: from the viewpoint of evolutionary genomics. Current Genomics, 13, 103–114. 23024602 10.2174/138920212799860706 PMC3308321

[evo14591-bib-0051] Niimura, Y. & Nei, M. (2005) Comparative evolutionary analysis of olfactory receptor gene clusters between humans and mice. Gene, 346, 13–21. 15716120 10.1016/j.gene.2004.09.025

[evo14591-bib-0052] Niimura, Y. , Matsui, A. & Touhara, K. (2014) Extreme expansion of the olfactory receptor gene repertoire in African elephants and evolutionary dynamics of orthologous gene groups in 13 placental mammals. Genome Research, 24, 1485–1496. 25053675 10.1101/gr.169532.113 PMC4158756

[evo14591-bib-0053] Paradis, E. , Claude, J. & Strimmer, K. (2004) APE: analyses of phylogenetics and evolution in R language. Bioinformatics (Oxford, England), 20, 289–290. 14734327 10.1093/bioinformatics/btg412

[evo14591-bib-0054] Potter, J.H.T. , Davies, K.T.J. , Yohe, L.R. , Sanchez, M.K.R. , Rengifo, E.M. , Struebig, M. , Warren, K. , Tsagkogeorga, G. , Lim, B.K. , Dos Reis, M. , et al. (2021) Dietary diversification and specialization in neotropical bats facilitated by early molecular evolution. Molecular Biology and Evolution, 38, 3864–3883. 34426843 10.1093/molbev/msab028 PMC8382914

[evo14591-bib-0055] Rodriguez, I. (2013) Singular expression of olfactory receptor genes. Cell, 155, 274–277. 24120129 10.1016/j.cell.2013.09.032

[evo14591-bib-0056] Rojas, D. , Warsi, O.M. & Dávalos, L.M. (2016) Bats (Chiroptera: Noctilionoidea) challenge a recent origin of extant neotropical diversity. Systematic Biology, 65, 432–448. Oxford University Press. 26865275 10.1093/sysbio/syw011

[evo14591-bib-0057] Rojas, D. , Pereira, M.J. , Fonseca, C. & Davalos, L.M. (2018) Eating down the food chain: generalism is not an evolutionary dead end for herbivores. Ecology Letters, 21, 402–410. 29341410 10.1111/ele.12911

[evo14591-bib-0058] Ruf, I. (2014) Comparative anatomy and systematic implications of the turbinal skeleton in Lagomorpha (Mammalia). The Anatomical Record, 297, 2031–2046. 25312363 10.1002/ar.23027

[evo14591-bib-0059] Santana, S.E. , Kaliszewska, Z.A. , Leiser‐Miller, L.B. , Lauterbur, M.E. , Arbour, J.H. , Dávalos, L.M. & Riffell, J.A. (2021) Fruit odorants mediate co‐specialization in a multispecies plant–animal mutualism. Proceedings of the Royal Society B: Biological Sciences, 288, 20210312. 10.1098/rspb.2021.0312 PMC8354748 34375556

[evo14591-bib-0060] Shi, J.J. & Rabosky, D.L. (2015) Speciation dynamics during the global radiation of extant bats. Evolution (N. Y), 69, 1528–1545. 10.1111/evo.12681 25958922

[evo14591-bib-0061] Simpson, G.G. (1953) The major features of evolution. Simon and Schuster, New York.

[evo14591-bib-0062] Smith‐Unna, R. , Boursnell, C. , Patro, R. , Hibberd, J.M. & Kelly, S. (2016) TransRate: reference‐free quality assessment of *de novo* transcriptome assemblies. Genome Research, 26, 1134–1144. 27252236 10.1101/gr.196469.115 PMC4971766

[evo14591-bib-0063] Thies, W. , Kalko, E.K.V. & Schnitzler, H.‐U. (1998) The roles of echolocation and olfaction in two Neotropical fruit‐eating bats, *Carollia perspicillata* and *C. castanea*, feeding on *Piper* . Behavioral Ecology and Sociobiology, 42, 397–409.

[evo14591-bib-0064] Treloar, H.B. , Miller, A.M. , Ray, A. & Greer, C.A. (2010) Development of the olfactory system. Pp. 131–155 *in* A. Menini , ed. The Neurobiology of Olfaction. CRC Press, Boca Raton, FL. 21882426

[evo14591-bib-0065] van Valkenburgh, B. , Pang, B. , Bird, D. , Curtis, A. , Yee, K.K. , Wysocki, C. & Craven, B.A. (2014) Respiratory and olfactory turbinals in feliform and caniform carnivorans: the influence of snout length. The Anatomical Record, 297, 2065–2079. 25312365 10.1002/ar.23026

[evo14591-bib-0079] VGstudio Max 3.3 . (2014) Heidelberg, Germany.

[evo14591-bib-0066] Wheeler, T.J. & Eddy, S.R. (2013) nhmmer: DNA homology search with profile HMMs. Bioinformatics (Oxford, England), 29, 2487–2489. 23842809 10.1093/bioinformatics/btt403 PMC3777106

[evo14591-bib-0067] Whelan, S. , Allen, J.E. , Blackburne, B.P. & Talavera, D. (2015) ModelOMatic: fast and automated model selection between RY, nucleotide, amino acid, and codon substitution models. Systematic Biology, 64, 42–55. 25209223 10.1093/sysbio/syu062

[evo14591-bib-0068] Yohe, L.R. & Brand, P. (2018) Evolutionary ecology of chemosensation and its role in sensory drive. Current Zoology, 64, 525–533. 30108633 10.1093/cz/zoy048 PMC6084603

[evo14591-bib-0069] Yohe, L.R. , Velazco, P.M. , Rojas, D. , Gerstner, B.E. , Simmons, N.B. & Davalos, L.M. (2015) Bayesian hierarchical models suggest oldest known plant‐visiting bat was omnivorous. Biology Letters, 11, 1–5. 10.1098/rsbl.2015.0501 PMC4685534 26559512

[evo14591-bib-0070] Yohe, L.R. , Hoffmann, S. & Curtis, A. (2018) Vomeronasal and olfactory structures in bats revealed by diceCT clarify genetic evidence of function. Frontiers in Neuroanatomy, 12, 1–13. 29867373 10.3389/fnana.2018.00032 PMC5953337

[evo14591-bib-0071] Yohe, L.R. , Devanna, P. , Davies, K.T. , Potter, J.H. , Rossiter, S.J. , Teeling, E.C. , Vernes, S. & Dávalos, L.M. (2019a) Tissue collection of bats for ‐omics analyses and primary cell culture. Journal of Visualized Experiments, 152, e59505. 10.3791/59505 31710024

[evo14591-bib-0072] Yohe, L.R. , Liu, L. , Dávalos, L.M. & Liberles, D.A. (2019b) Protocols for the molecular evolutionary analysis of membrane protein gene duplicates. Pp. 49–62 *in* T. Sikosek , ed. Computational methods in protein evolution. Springer New York, New York. 10.1007/978-1-4939-8736-8_3 30298391

[evo14591-bib-0073] Yohe, L.R. , Davies, K.T. , Simmons, N.B. , Sears, K.E. , Dumont, E.R. , Rossiter, S.J. & Dávalos, L.M. (2020a) Evaluating the performance of targeted sequence capture, RNA‐Seq, and degenerate‐primer PCR cloning for sequencing the largest mammalian multigene family. Molecular Ecology Resources, 20, 140–153. 31523924 10.1111/1755-0998.13093

[evo14591-bib-0074] Yohe, L.R. , Fabbri, M. , Hanson, M. & Bhullar, B.‐A.S. (2020b) Olfactory receptor gene evolution is unusually rapid across Tetrapoda and outpaces chemosensory phenotypic change. Current Zoology, 66, 505–514. 34484311 10.1093/cz/zoaa051 PMC7750991

[evo14591-bib-0075] Yohe, L.R. , Leiser‐Miller, L.B. , Kaliszewska, Z.A. , Donat, P. , Santana, S.E. & Dávalos, L.M. (2021) Diversity in olfactory receptor repertoires is associated with dietary specialization in a genus of frugivorous bat. G3 Genes|Genomes|Genetics, 11, jkab260. 34568918 10.1093/g3journal/jkab260 PMC8473985

[evo14591-bib-0076] Zou, D.‐J. , Chesler, A. & Firestein, S. (2009) How the olfactory bulb got its glomeruli: a just so story? Nature Reviews. Neuroscience, 10, 611–618. 19584894 10.1038/nrn2666

