# Supplementary material for: Ecological constraints on highly evolvable olfactory receptor genes and morphology in neotropical bats
Source: Evolution. 2022 Aug 30;76(10):2347–60. doi: 10.1111/evo.14591 (PMC9804929; doi:10.1111/evo.14591)
Supplement: Supplementary file 1 — Supplementary Information [file EVO-76-2347-s001.pdf]

Supplementary Information for
Ecological constraints on highly evolvable olfactory receptor genes and morphology in neotropical bats

Supplementary Information Text

Supplementary Methods and Results

Statistical analyses of evolutionary rates:

To explore more deeply the rates of trait evolution for the surface area of the olfactory epithelium, we also tested how Ornstein-Uhlenbeck models performed relative to our regressions presented in the main text. For allometric models, surface area, body mass, or both may evolve though stabilizing or directional selection (or neutrally) (1, 2) or under little to no selection, but standard phylogenetic regressions assume a Brownian motion (BM) model for residuals. Accounting for directional selection requires enriching the BM model of evolution with one or more optima and a rate of directional evolution alpha, generating an Ornstein-Uhlenbeck process (3). For the bivariate case the allometry could apply to the entire sample with one optimum (intercept) and one slope, the optima could vary while the slope could apply to the whole sample, or both intercepts and slopes could vary across groups. The bayou R package implements Bayesian reversible-jump Markov chain Monte Carlo process that enables inferring the optima based on the data. Additionally, we evaluated one model with different optima for animal– or plant–plant eating species. Bayou models were compared using the stepping–stone procedure to estimate the likelihood.

Ornstein-Uhlenbeck models, however, can reveal a low rate of directional evolution alpha, which makes it feasible to model allometric scaling assuming BM. Therefore, we also estimated allometric scaling parameters using standard phylogenetic regressions. Evolutionary allometric models tend to assume a single intercept and slope explains the relationship of a given trait to log mass, but adaption yields different intercepts, and the allometric slope may not be uniform across clades. Analyses of directional evolution of surface area as a function of body mass identified a multi-optimum, single slope model as the one with the highest marginal likelihood (-30.8) compared to others (<-45.8, Table S11). Posterior parameter estimates summarized in Table S12, however, show weak support for multiple optima and estimates of the directional evolution parameter alpha were lower than the random walk parameter σ^2. Inspection of the posterior probabilities for change in optima in the phylogeny revealed a >0.50 probability in the ancestor of mormoopids (Fig. S6), four optima with a >0.25 probability and shifts in eleven branches with a posterior probability >0.1. In the scenario with many optima (mean = 6; lower = 1, upper = 12) and corresponding shifts from one optimum to another, shifts are distributed across the tree and unrelated to plant-eating. There was no statistically significant separation by diet. Ultimately, our data set is too small to establish meaningful relationships or optima and there are only 1-2 shifts to plant visiting in our tree. However, this exercise helped to enlighten and confirm how mormoopids continue to confound phylogenetic comparative methods in bats.

Fig. S1. Phylogeny of cumulative taxa used in this study. Iodine-stained μCT-scans were used to reconstruct olfactory epithelium of different turbinates. RNA-seq of the main olfactory epithelium was used to identify protein-coding sequences of expressed olfactory receptors. For some species, different food resources are equally abundant (e.g. *Phyllostomus hastatus*). In the "Plant" column, circles represent how each species was coded in ANOVA analyses. Coding was determined from the continuous values calculated from Rojas *et al.*, (2018), such that negative values represent diets that include more plant resources (black circles), while positive values indicate diets that include more animal resources (white circles). Grey bars are the number of intact olfactory receptors identified in the RNA-seq data.

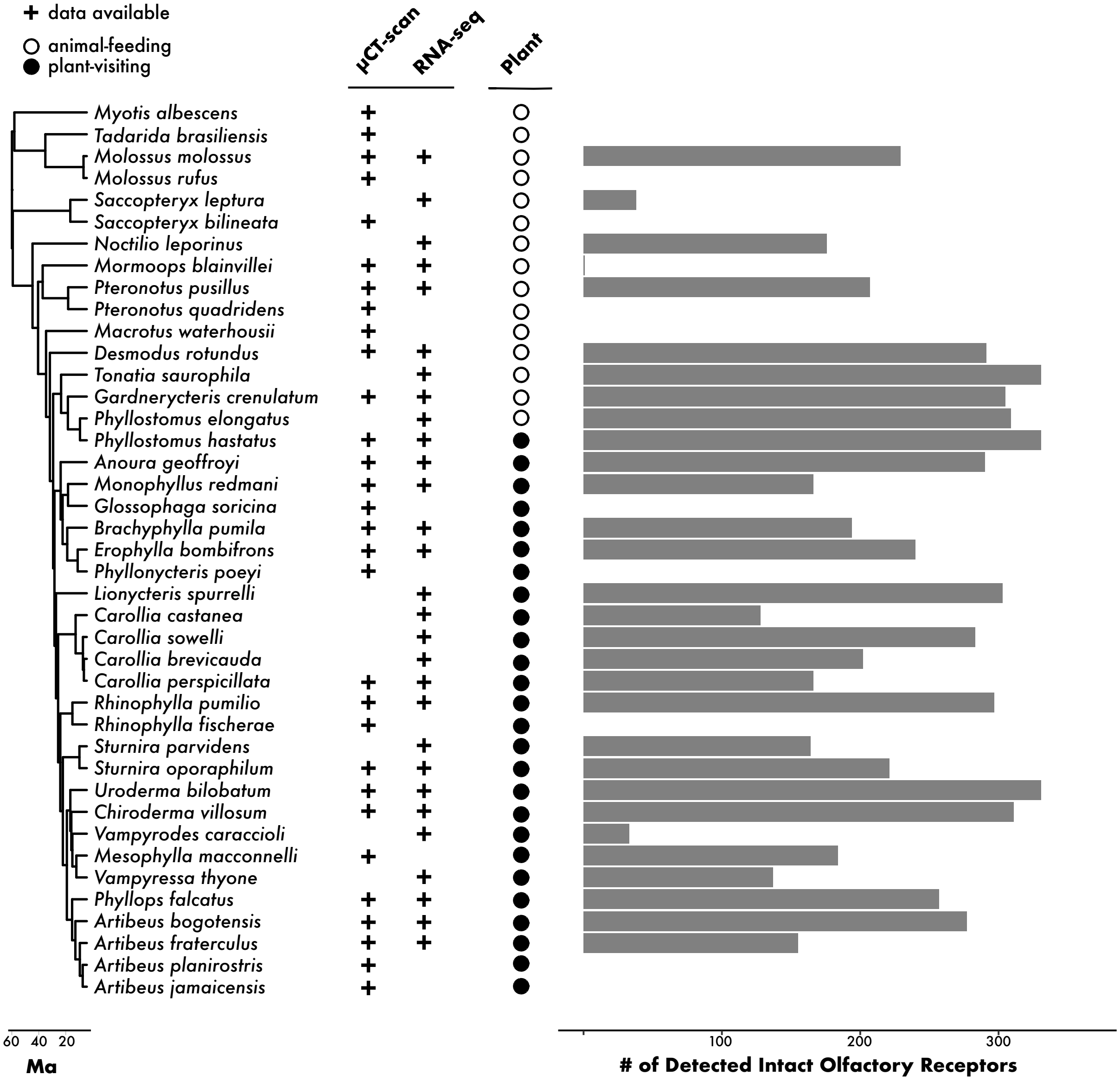

Fig. S2. Parameter estimates of MCMCglmm including the mormoopids, testing for a relationship of olfactory epithelium surface area and body mass, explained by diet. Open circles denote posterior estimates overlap with zero; grey circles denote 95% credible intervals overlap with zero; and black circles indicate the entire posterior distribution is above or below zero.

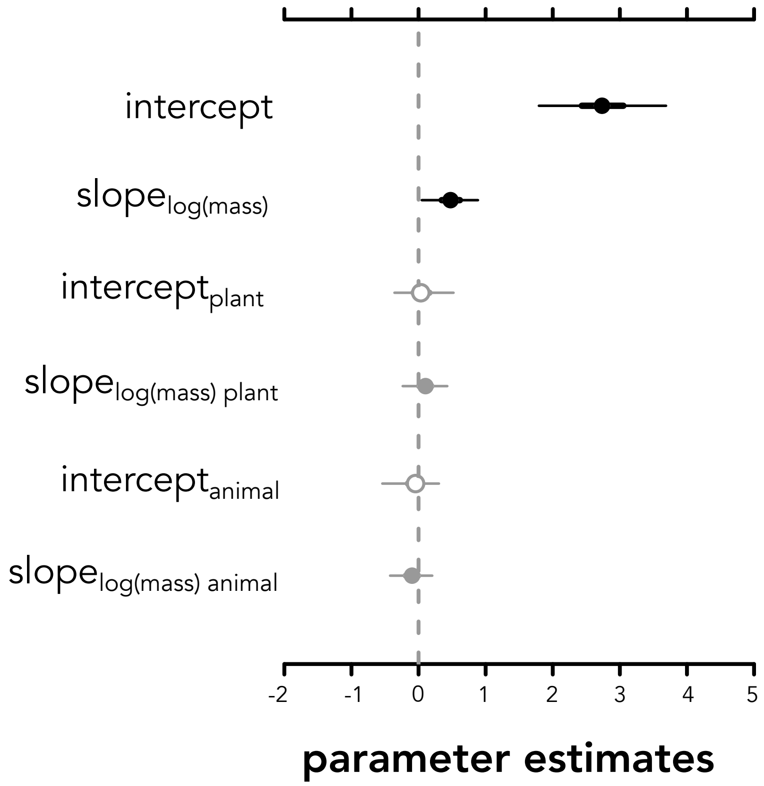

Fig. S3. RNA integrity number (RIN) versus number of distinct and intact olfactory receptors recovered from the transcriptomes of the main olfactory epithelium.

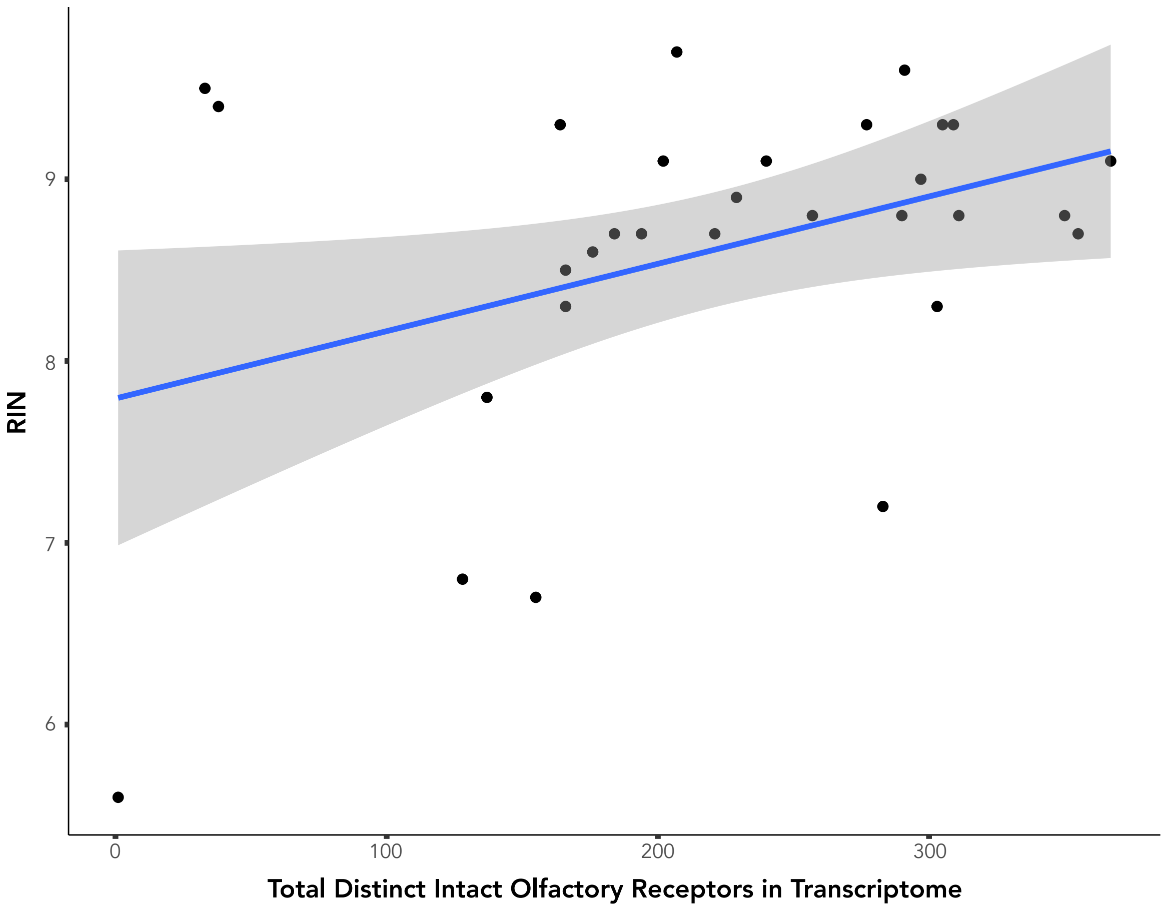

Fig S4. Posterior predictive check of model fit using estimated parameters from the observed data (left) to predict response (right) of the molecular-only MCMCglmm model. This model included mormoopids but results were similar for both.

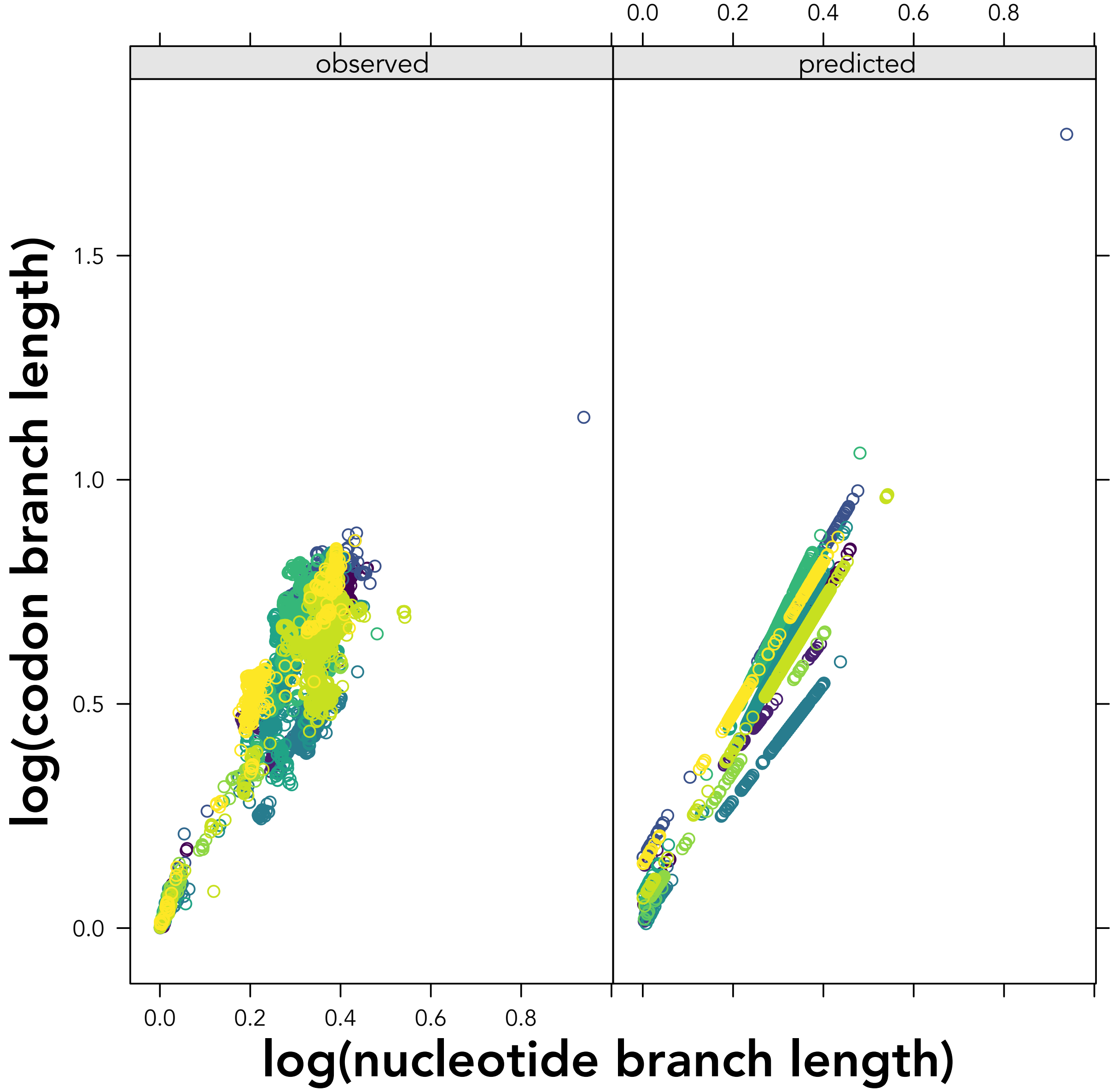

Fig. S5. In models including mormoopids, the best-fit, single-response model of codon rates had different nucleotide rate slopes by gene subfamily (DIC: -12521), but covaried neither with body mass (mean slope = 0.00, lower = -0.18, upper = 0.17), nor with olfactory epithelium surface area (mean slope = 0.00, lower = -0.22, upper = 0.19). The best multi-response model (DIC: -37342) had qualitatively similar results, with only a weak trend for log body mass of plant-eating bats relating to codon rates (mean slope = -0.0038, lower = -0.0128, upper = 0.0042).

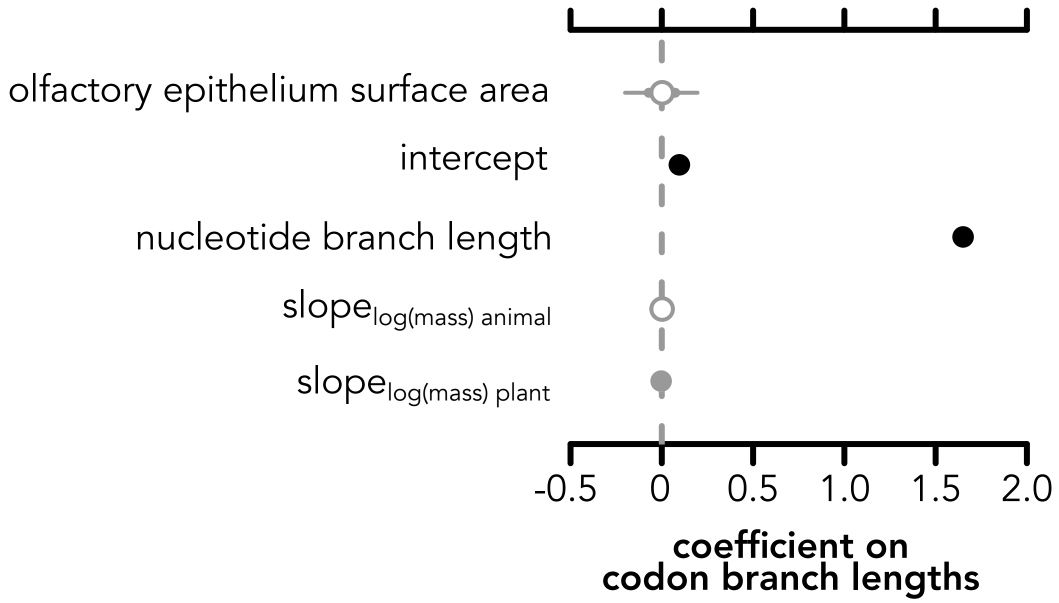

Fig. S6. Bayou outputs for regime shift (β) with greater than 0.5 posterior probability, showing most probably shift (decrease) in surface area of olfactory epithelium.

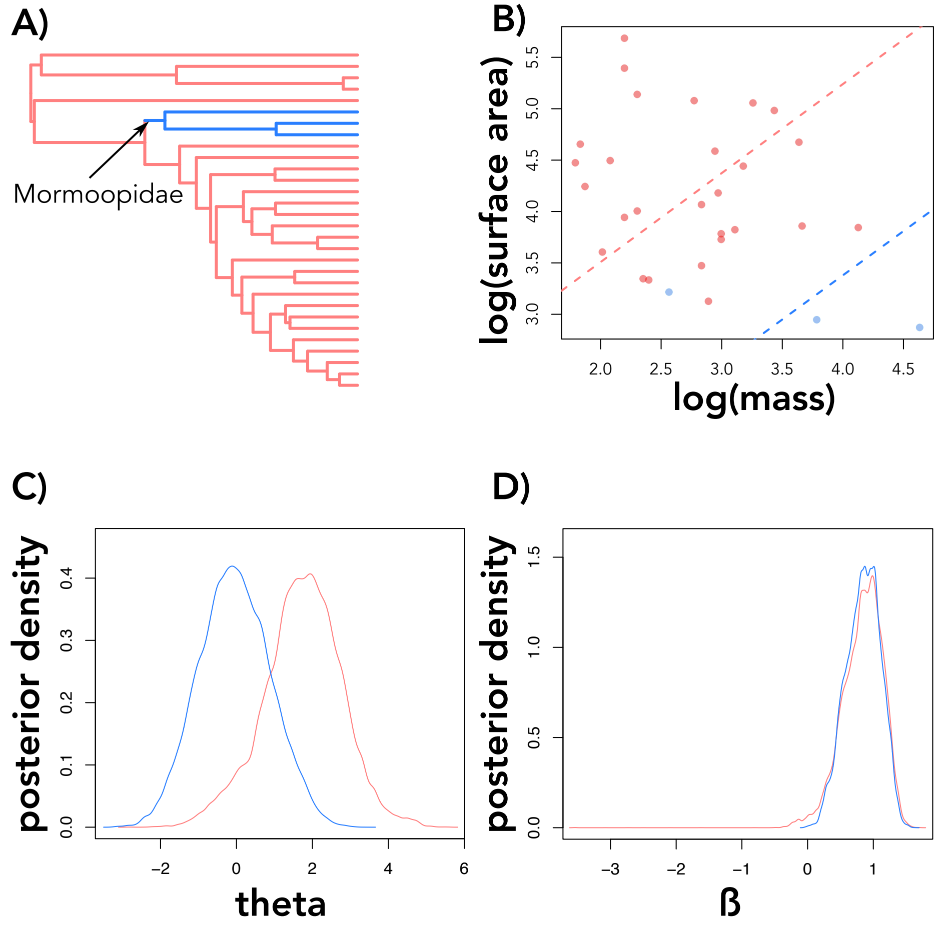

Table S1. Field data for specimens used in morphological analyses. Specimens were fixed in 4% paraformaldehyde, stained in 10% Lugol's iodide solution, and μCT-scanned. The asterisk (*) indicates species in which transcriptomic data is also available.

Family	Species	Field Number	Locality	Date Captured	Sex
Emballonuridae	*Saccopteryx bilineata*	PE160	Jenaro Herrera, Peru	May 22 2015	M
Vespertilionidae	*Myotis albescens*	PE008	Roca Rajada, Suyo, Peru	May 13 2015	M
Molossidae	*Tadarida brasiliensis*	DR028	Ebano Verde, Dominican Republic	Feb 7 2014	M
Molossidae	*Molossus rufus*	PE016	Roca Rajada, Suyo, Peru	May 13 2015	M
Molossidae	*Molossus molossus**	PE156	Jenaro Herrera, Peru	May 22 2015	M
Mormoopidae	*Pteronotus pusillus**	DR046	Jaragua, Dominican Republic	Feb 8 2014	M
Mormoopidae	*Pteronotus quadridens*	DR098	Cotui, Dominican Republic	Feb 12 2014	M
Mormoopidae	*Mormoops blainvillei**	DR092	Cotui, Dominican Republic	Feb 12 2014	M
Phyllostomidae	*Macrotus waterhousii*	DR059	Jaragua, Dominican Republic	Feb 9 2014	M
Phyllostomidae	*Desmodus rotundus**	PE063	San Antonio, Faique, Peru	May 16 2015	M
Phyllostomidae	*Phyllostomus hastatus**	PE088	Jenaro Herrera, Peru	May 20 2015	M
Phyllostomidae	*Gardnerycteris crenulatum**	PE136	Jenaro Herrera, Peru	May 21 2015	M
Phyllostomidae	*Monophyllus redmani**	DR022	Salto de Socoa, Dominican Republic	Feb 4 2014	M
Phyllostomidae	*Glossophaga soricina*	PE067	Jenaro Herrera, Peru	May 18 2015	M
Phyllostomidae	*Anoura geoffroyi**	PE040	San Cristobal, Faique, Peru	May 15 2015	M
Phyllostomidae	*Brachyphylla pumila**	DR235	Dominican Republic	Feb 2015	M
Phyllostomidae	*Erophylla bombifrons**	DR138	Dominican Republic	Feb 2015	M
Phyllostomidae	*Phyllonycteris poeyi*	DR166	Dominican Republic	Feb 2015	M
Phyllostomidae	*Rhinophylla fischerae*	PE101	Jenaro Herrera, Peru	May 20 2015	M
Phyllostomidae	*Rhinophylla pumilio**	PE098	Jenaro Herrera, Peru	May 20 2015	M
Phyllostomidae	*Carollia perspicillata**	PE068	Jenaro Herrera, Peru	May 18 2015	M
Phyllostomidae	*Sturnira oporaphilum**	PE018	San Antonio, Faique, Peru	May 14 2015	M
Phyllostomidae	*Mesophylla macconnelli**	PE092	Jenaro Herrera, Peru	May 20 2015	M
Phyllostomidae	*Phyllops falcatus**	DR065	Jaragua, Dominican Republic	Feb 9 2014	M
Phyllostomidae	*Chiroderma villosum*	PE170	Jenaro Herrera, Peru	May 22 2015	F
Phyllostomidae	*Artibeus planirostris*	PE076	Jenaro Herrera, Peru	May 19 2015	M
Phyllostomidae	*Artibeus fraterculus**	PE004	Roca Rajada, Suyo, Peru	May 13 2015	M
Phyllostomidae	*Artibeus bogotensis**	PE126	Jenaro Herrera, Peru	May 20 2015	M
Phyllostomidae	*Artibeus jamaicensis*	DR151	Dominican Republic	Feb 2015	F
Phyllostomidae	*Uroderma bilobatum**	PE090	Jenaro Herrera, Peru	May 20 2015	M

Table S2. Specimen sampling and locality information for those used in RNA-seq analyses. AMNH-M are samples deposited in the American Museum of Natural History Mammalogy collection. The remaining tissues are a part of the collection housed at Stony Brook University.

Family	Species	Field Number	Locality	Date Captured	Sex
Emballonuridae	*Saccopteryx leptura*	PE157	Jenaro Herrera, Peru	May 22 2015	M
Molossidae	*Molossus molossus*	PE078	Jenaro Herrera, Peru	May 19 2015	M
Noctilionidae	*Noctilio leporinus*	DR101	Cotui, Dominican Republic	Feb 12 2014	F
Mormoopidae	*Mormoops blainvillei*	DR091	Cotui, Dominican Republic	Feb 12 2014	M
Mormoopidae	*Pteronotus pusillus* (formerly *parnellii*)	DR038	Jaragua, Dominican Republic	Feb 8 2014	M
Phyllostomidae	*Desmodus rotundus*	AMNH-M 278722	Ka'Kabish Archaeological Project, Belize	Apr 30 2014	M
Phyllostomidae	*Phyllostomus hastatus*	PE091	Jenaro Herrera, Peru	May 20 2015	M
Phyllostomidae	*Phyllostomus elongatus*	PE109	Jenaro Herrera, Peru	May 20 2015	F
Phyllostomidae	*Gardnerycteris crenulatum* (formerly *Mimon*)	PE095	Jenaro Herrera, Peru	May 20 2015	M
Phyllostomidae	*Tonatia saurophila*	PE084	Jenaro Herrera, Peru	May 20 2015	M
Phyllostomidae	*Monophyllus redmani*	DR013	Salto de Socoa, Dominican Republic	Feb 4 2014	M
Phyllostomidae	*Anoura geoffroyi*	PE023	San Antonio, Faique, Peru	May 14 2014	M
Phyllostomidae	*Brachyphylla pumila*	DR122	La Chepa, Dominican Republic	Feb 13 2014	M
Phyllostomidae	*Erophylla bombifrons*	DR086	Cueva IV Pomier, Dominican Republic	Feb 11 2014	M
Phyllostomidae	*Lionycteris spurrelli*	PE171	Jenaro Herrera, Peru	May 22 2015	M
Phyllostomidae	*Rhinophylla pumilio*	PE139	Jenaro Herrera, Peru	May 21 2015	M
Phyllostomidae	*Carollia brevicauda*	PE111	Jenaro Herrera, Peru	May 20 2015	M
Phyllostomidae	*Carollia castanea*	LS084	La Selva, Costa Rica	Aug 4 2017	M
Phyllostomidae	*Carollia sowelli*	LS073	La Selva, Costa Rica	Aug 4 2017	M
Phyllostomidae	*Carollia perspicillata*	LS070	La Selva, Costa Rica	Aug 4 2017	M
Phyllostomidae	*Phyllops falcatus*	DR003	Salto de Socoa, Dominican Republic	Feb 4 2014	F
Phyllostomidae	*Chiroderma villosum*	PE152	Jenaro Herrera, Peru	May 21 2015	M
Phyllostomidae	*Mesophylla macconnelli*	PE150	Jenaro Herrera, Peru	May 21 2015	M
Phyllostomidae	*Vampyressa thyone*	AMNH-M 278709	Orange Walk District, Lamanai, Belize	Apr 29 2014	M
Phyllostomidae	*Vampyrodes caraccioli*	PE153	Jenaro Herrera, Peru	May 21 2015	M
Phyllostomidae	*Artibeus bogotensis*	PE174	Jenaro Herrera, Peru	May 23 2015	M
Phyllostomidae	*Artibeus fraterculus*	PE005	Roca Rojada, Suyo, Peru	May 13 2015	M
Phyllostomidae	*Sturnira parvidens* (formerly *lilium*)	AMNH-M 278693	Lamanai Archaeological Reserve (Ball Court), Lamanai, Belize	Apr 28 2014	M
Phyllostomidae	*Sturnira oporaphilum* (formerly *ludovici*)	PE019	San Antonio, Faique, Peru	May 14 2015	M
Phyllostomidae	*Uroderma bilobatum*	PE175	Jenaro Herrera, Peru	May 23 2015	M

Table S3. μCT-scan parameters for morphological analyses. The asterisk (*) indicates scans that were from a previously published study (6), in which a 0.1mm copper filter was used. DiceCT scans are available in MorphoSource under ProjectID ID: 000393378 and the ARK IDs are provided in the table below.

Specimen	Species	kV	μA	Voxelsize (mm)	MorphoSource ID
PE160	*Saccopteryx bilineata*	80	70	0.01018564	ark:/87602/m4/393533
PE008	*Myotis albescens*	81	68	0.00965001	ark:/87602/m4/393605
DR028	*Tadarida brasiliensis*	80	68	0.01157217	ark:/87602/m4/393759
PE016	*Molossus rufus*	80	69	0.01409494	ark:/87602/m4/393771
PE156	*Molossus molossus*	81	69	0.01103854	ark:/87602/m4/393783
DR046	*Pteronotus pusillus* (formerly *parnellii*)	87	103	0.01050018	ark:/87602/m4/393923
DR098	*Pteronotus quadridens*	81	66	0.00853105	ark:/87602/m4/393837
DR092	*Mormoops blainvillei*	83	68	0.01104236	ark:/87602/m4/393934
DR059	*Macrotus waterhousii*	80	73	0.0186078	ark:/87602/m4/393939
PE063	*Desmodus rotundus*	103	69	0.01639554	ark:/87602/m4/393944
PE088	*Phyllostomus hastatus*	99	71	0.02052979	ark:/87602/m4/393949
PE136	*Gardnerycteris crenulatum* (formerly *Mimon*)	82	68	0.01274507	ark:/87602/m4/394460
DR022	*Monophyllus redmani*	80	68	0.01243628	ark:/87602/m4/393961
PE040	*Anoura geoffroyi*	80	69	0.01417887	ark:/87602/m4/393983
PE067	*Glossophaga soricina*	80	69	0.01223687	ark:/87602/m4/393966
DR235*	*Brachyphylla pumila*	130	150	0.0207	ark:/87602/m4/395147
DR138*	*Erophylla bombifrons*	110	130	0.0196	ark:/87602/m4/395190
DR166*	*Phyllonycteris poeyi*	150	120	0.0194	ark:/87602/m4/395166
PE101	*Rhinophylla fischerae*	81	67	0.00975981	ark:/87602/m4/394474
PE098	*Rhinophylla pumilio*	81	68	0.01077508	ark:/87602/m4/393999
PE068	*Carollia perspicillata*	81	67	0.01536867	ark:/87602/m4/394479
PE018	*Sturnira oporaphilum* (formerly *ludovici*)	80	69	0.01428175	ark:/87602/m4/394687
PE092	*Mesophylla macconnelli*	81	68	0.00928052	ark:/87602/m4/394008
DR065	*Phyllops falcatus*	80	69	0.01276184	ark:/87602/m4/394146
PE170	*Chiroderma villosum*	80	67	0.01593133	ark:/87602/m4/394171
PE076	*Artibeus planirostris*	100	69	0.01678076	ark:/87602/m4/394206
PE004	*Artibeus fraterculus*	99	70	0.01587278	ark:/87602/m4/394304
PE126	*Artibeus bogotensis*	81	67	0.01057702	ark:/87602/m4/394390
DR151*	*Artibeus jamaicensis*	150	120	0.0196	ark:/87602/m4/395127
PE090	*Uroderma bilobatum*	81	67	0.01357078	ark:/87602/m4/394390

Table S4. Read count is combined paired read number after cleaning and trimming (i.e., the number of reads that went into the Oyster River Protocol assembly). *Desmodus rotundus* raw reads have already been deposited to GenBank and published from a previous study (5). Mass is of RNA. All sequence read archives are deposited into BioProject PRJNA563501on NCBI.

Species	Mass (μg)	RIN	Company	Platform	Read Length	Read count	Biosample	SRA
Saccopteryx leptura	0.98	9.4	BGI	HiSeq 4000	100	73,848,364	SAMN28423144	SRR19391913
Molossus molossus	0.53	8.9	BGI	HiSeq 4000	100	73,965,692	SAMN28423151	SRR19391912
Noctilio leporinus	1.4	8.6	BGI	HiSeq 4000	100	73,722,246	SAMN28423239	SRR19391901
Mormoops blainvillei	0.82	5.6	AZ	HiSeq 2500	100	64,426,826	SAMN28423241	SRR19391891
Pteronotus pusillus (formerly *parnellii*)	3.29	9.7	BGI	HiSeq 4000	100	73,599,542	SAMN28423253	SRR19391890
Desmodus rotundus	1.09	9.6	BGI	HiSeq 4000	100	73,492,622	SAMN12675092	SRR8878915
Phyllostomus hastatus	2.16	9.1	BGI	HiSeq 4000	100	73,641,072	SAMN28423152	SRR19391889
Phyllostomus elongatus	1.64	9.3	BGI	HiSeq 4000	100	73,888,848	SAMN28423153	SRR19391888
Gardnerycteris crenulatum (formerly *Mimon*)	0.29	9.3	BGI	HiSeq 4000	100	73,478,232	SAMN28423154	SRR19391887
Tonatia saurophila	1.91	8.8	BGI	HiSeq 4000	100	73,753,554	SAMN28423155	SRR19391886
Monophyllus redmani	0.157	8.3	AZ	HiSeq 2500	100	58,099,750	SAMN28423430	SRR19391885
Anoura geoffroyi	1.03	8.8	BGI	HiSeq 4000	100	73,754,230	SAMN28425429	SRR19391911
Brachyphylla pumila	3.22	8.7	BGI	HiSeq 4000	100	73,649,738	SAMN28425589	SRR19391910
Erophylla bombifrons	0.97	9.1	BGI	HiSeq 2000	90	73,186,614	SAMN28425590	SRR19391909
Lionycteris spurrelli	1.1	8.3	BGI	HiSeq 4000	100	73,664,158	SAMN28423180	SRR19391908
Rhinophylla pumilio	3.72	9.0	BGI	HiSeq 4000	100	74,055,080	SAMN28423181	SRR19391907
Carollia brevicauda	1.98	9.1	BGI	HiSeq 4000	100	73,523,188	SAMN12675087	SRR19391906
Carollia castanea	1.56	6.8	Novogene	NovaSeq 6000	150	55,330,326	SAMN12675090	SRR19391905
Carollia sowelli	0.45	7.2	Novogene	NovaSeq 6000	150	61,151,460	SAMN12675089	SRR19391904
Carollia perspicillata	2.24	8.5	Novogene	NovaSeq 6000	150	53,094,822	SAMN12675088	SRR19391903
Phyllops falcatus	2.03	8.8	BGI	HiSeq 4000	100	73,553,010	SAMN28425673	SRR19391902
Chiroderma villosum	2.12	8.8	BGI	HiSeq 4000	100	73,722,350	SAMN28423182	SRR19391900
Mesophylla macconnelli	5.33	8.7	BGI	HiSeq 4000	100	73,654,484	SAMN28423183	SRR19391899
Vampyrodes caraccioli	8.9	9.5	BGI	HiSeq 4000	100	73,613,076	SAMN28423184	SRR19391898
Vampyressa thyone	0.95	7.8	BGI	HiSeq 2000	90	73,755,914	SAMN28453132	SRR19391897
Artibeus bogotensis	3.96	9.3	BGI	HiSeq 4000	100	69,514,000	SAMN28423186	SRR19391896
Artibeus fraterculus	1.96	6.7	BGI	HiSeq 4000	100	74,695,636	SAMN12675084	SRR19391895
Sturnira parvidens (formerly *lilium*)	1.25	9.3	BGI	HiSeq 2000	90	72,558,600	SAMN12675086	SRR19391894
Sturnira oporaphilum (formerly *ludovici*)	0.59	8.7	BGI	HiSeq 4000	100	73,955,198	SAMN12675085	SRR19391893
Uroderma bilobatum	4.58	8.7	BGI	HiSeq 4000	100	75,088,106	SAMN28423187	SRR19391892

Table S5. Summary of model parameters (and 95% high probability density interval) and deviance information criterion (DIC) for morphological regressions relating surface to mass, both in log scale. When partitioned, intercepts and/or slopes are by animal/plant diet. The best-fit model is in **bold**.

Model	intercept	intercept plant	slope	slope plant	DIC
Single intercept, single slope	2.6 (1.6, 3.6)	—	0.5 (0.2, 0.9)	—	60.9
Two intercepts, single slope	2.7 (1.2, 4.0)	2.8 (1.4, 4.2)	0.5 (0.05, 0.9)		53.4
**Two intercepts, two slopes**	**2.7 (1.2, 4.0)**	**2.8 (1.4, 4.2)**	**-0.1 (-0.4, 0.2)**	**0.1 (-0.2, 0.4)**	**52.6**

Table S6. ORF:Gene count is the number of open reading frames detected compared to the number of "genes" or contigs assembled. Mean length is in number of base pairs.

Species	ORF:Gene count	Unique Genes	Mean length	Good mapping	Bases uncov	Contigs uncov	Low cov	Segmented	Transrate Score	Transrate Optimum
Saccopteryx leptura	35,379: 212,005	626	711	0.90	0.03	0.01	0.71	0.08	0.48	0.57
Molossus molossus	31,772: 222,376	787	617	0.89	0.03	0.01	0.66	0.09	0.44	0.53
Noctilio leporinus	37,650: 248,557	707	699	0.92	0.04	0.02	0.80	0.07	0.52	0.59
Mormoops blainvillei	32,841: 400,658	1182	392	0.80	0.05	0.02	0.84	0.06	0.39	0.49
Pteronotus pusillus	41,491: 258,066	550	756	0.90	0.04	0.02	0.82	0.06	0.49	0.59
Desmodus rotundus	39,470: 255,295	564	733	0.91	0.04	0.01	0.79	0.07	0.51	0.59
Phyllostomus hastatus	41,259: 256,366	563	746	0.92	0.04	0.02	0.82	0.07	0.53	0.61
Phyllostomus elongatus	39,686: 229,723	739	753	0.92	0.04	0.01	0.78	0.07	0.51	0.60
Gardnerycteris crenulatum	38,169: 239,853	622	691	0.92	0.03	0.01	0.76	0.09	0.51	0.59
Tonatia saurophila	41,354: 288,237	830	709	0.93	0.04	0.02	0.84	0.06	0.56	0.63
Monophyllus redmani	35,403: 289,168	1054	475	0.84	0.07	0.03	0.80	0.06	0.44	0.55
Anoura geoffroyi	39,922: 238,939	919	757	0.93	0.04	0.02	0.82	0.06	0.54	0.61
Brachyphylla pumila	41,227: 267,268	535	746	0.92	0.04	0.02	0.81	0.07	0.52	0.60
Erophylla bombifrons	30,897: 246,685	460	632	0.92	0.02	0.01	0.86	0.03	0.60	0.66
Lionycteris spurrelli	38,935: 263,713	846	693	0.92	0.03	0.01	0.79	0.06	0.52	0.59
Rhinophylla pumilio	39,147: 254,100	483	706	0.93	0.04	0.02	0.82	0.07	0.53	0.62
Carollia brevicauda	31,357: 170,475	485	724	0.91	0.05	0.02	0.85	0.07	0.54	0.65
Carollia castanea	30,890: 164,372	682	649	0.93	0.07	0.03	0.83	0.05	0.22	0.65
Carollia sowelli	41,640: 327,035	1058	637	0.92	0.05	0.01	0.76	0.05	0.18	0.60

Carollia perspicillata	35,213: 245,794	663	645	0.92	0.04	0.02	0.79	0.05	0.16	0.59
Phyllops falcatus	39.830:238,280	550	755	0.93	0.04	0.02	0.81	0.07	0.53	0.62
Chiroderma villosum	38,195: 278,203	786	684	0.92	0.03	0.01	0.79	0.07	0.53	0.61
Mesophylla macconnelli	34,494: 180,541	706	742	0.92	0.04	0.01	0.68	0.11	0.48	0.58
Vampyrodes caraccioli	36,659: 200,806	527	764	0.93	0.04	0.02	0.83	0.06	0.53	0.61
Vampyressa thyone	33,041: 305,765	485	567	0.90	0.02	0.01	0.86	0.04	0.59	0.63
Artibeus bogotensis	37,367: 228,913	537	714	0.89	0.03	0.01	0.79	0.07	0.49	0.57
Artibeus fraterculus	33,538: 216,820	747	670	0.93	0.03	0.01	0.75	0.09	0.51	0.57
Sturnira parvidens	31,141: 240,315	488	625	0.92	0.02	0.01	0.84	0.03	0.59	0.64
Sturnira oporaphilum	32,426: 206,895	671	667	0.91	0.03	0.01	0.74	0.09	0.47	0.57
Uroderma bilobatum	37,635: 199,053	927	783	0.93	0.04	0.02	0.78	0.08	0.51	0.60

Table S7. Number of intact olfactory receptors for each subfamily identified in the main olfactory epithelium transcriptome. An intact gene was determined to have an open reading frame of greater 650bp.

Species	OR 51	OR 52	OR 55	OR 56	OR 1/3/7	OR 2/13	OR 4	OR 5/8/9	OR 6	OR 10	OR 11	OR 12	OR 14	Total
Saccopteryx leptura	1	1	0	1	4	7	6	7	2	6	3	0	0	38
Molossus molossus	17	25	1	1	40	26	13	58	19	22	7	0	0	229
Noctilio leporinus	15	22	0	2	30	16	10	53	12	13	2	0	1	176
Mormoops blainvillei	0	0	0	0	0	0	0	1	0	0	0	0	0	1
Pteronotus pusillus	18	4	0	3	51	27	12	59	16	13	4	0	0	207
Desmodus rotundus	28	28	0	3	55	22	19	91	23	17	3	1	1	291
Phyllostomus hastatus	38	40	0	5	55	40	34	97	28	24	5	0	1	367
Phyllostomus elongatus	36	29	0	3	40	24	40	82	24	23	6	1	1	309
Gardnerycteris crenulatum	33	25	0	4	40	35	26	87	32	15	7	0	1	305
Tonatia saurophila	43	41	1	4	49	30	40	82	31	22	7	0	0	350
Monophyllus redmani	11	10	1	1	31	35	15	30	8	19	4	1	0	166
Anoura geoffroyi	30	30	0	7	46	42	23	67	19	18	6	1	1	290
Brachyphylla pumila	6	9	3	2	40	26	8	67	17	11	3	0	2	194
Erophylla bombifrons	26	21	2	1	44	21	16	65	16	17	9	1	1	240
Lionycteris spurrelli	36	29	0	4	42	25	34	84	15	22	11	1	0	303
Rhinophylla pumilio	22	21	0	3	53	34	31	88	11	24	10	0	0	297
Carollia brevicauda	20	15	1	3	32	16	26	50	11	21	7	0	0	202
Carollia castanea	8	2	0	0	29	15	18	41	3	9	3	0	0	128
Carollia sowelli	34	25	0	3	45	37	25	70	12	23	8	0	1	283
Carollia perspicillata	15	12	2	0	28	19	22	47	7	11	3	0	0	166
Phyllops falcatus	24	14	0	4	44	35	31	62	16	19	7	1	0	257
Chiroderma villosum	15	25	0	4	66	39	32	75	16	26	11	2	0	311
Mesophylla macconnelli	14	11	0	4	40	18	16	46	16	14	5	0	0	184
Vampyressa thyone	0	2	0	0	27	26	14	40	12	10	6	0	0	137
Vampyrodes caraccioli	1	0	0	0	10	3	1	14	1	3	0	0	0	33

Artibeus bogotensis	29	21	1	4	42	28	35	73	15	22	7	0	0	277
Artibeus fraterculus	1	3	0	0	32	31	18	34	16	14	5	0	1	155
Sturnira parvidens	8	9	0	2	28	18	22	40	16	15	4	1	1	164
Sturnira oporaphilum	6	9	0	3	29	35	36	56	16	25	5	0	1	221
Uroderma bilobatum	25	26	1	6	71	56	32	86	21	22	7	1	1	355

Table S8. Number of intact sequences for each olfactory receptor subfamily. Alignment length and percent identity was calculated from nucleotide alignments based on the transAlign algorithm. The models of evolution estimated for each alignment were determined by ModelOMatic v.1.01 and applied to each alignment for tree inference in IQ-TREE. Data in this table refer to the alignment with the root, but with stop codons for all sequences removed.

OR subfamily	# sequences	Alignment Length (bp)	% pairwise identity	Codon Model	Nucleotide Model
Class I					
OR 51	560	1,386	59.3	Codon F1X4+4dG	HKY+4dg
OR 52	511	1,314	58.2	Codon EQU+4dG	GTR+4dG
OR 55	10	1,080	80.8	Codon F64+4dG	GTR+4dG
OR 56	78	1,125	72.5	Codon F3X4+4dG	K2P+4dG
Class II					
OR 1/3/7	1,154	1,584	62.7	Codon EQU+4dG	GTR+4dG
OR 2/13	787	1,614	57.0	Codon EQU+4dG	GTR+4dG
OR 4	657	1,647	60.1	Codon EQU+4dG	HKY+4dG
OR 5/8/9	1,753	1,989	56.8	Codon EQU+4dG	K2P+4dG
OR 6	451	1,461	59.7	Codon F3X4+4dG	K2P+4dG
OR 10	501	1,335	54.6	Codon F1X4+4dG	GTR+4dG
OR 11	164	1,293	66.2	Codon EQU+4dG	GTR+4dG
OR 12	12	1,071	85.1	Codon F3X4	GTR+4dG
OR 14	15	1,077	83.9	Codon F3X4+4dG	GTR+4dG

Table S9. Deviance information criterion (DIC) values from hierarchical Bayesian phylogenetic regressions of codon lengths as a function of nucleotide lengths including mormoopids. The best-fit model is in **bold**.

Model	DIC
Single intercept, single slope	-11402.14
Single intercept, two slopes (animal/plant)	-11433.39
Single intercept, many slopes (diet categories)	-11428.93
**Single intercept, many slopes (*OR* gene family)**	**-18084.79**
Single intercept, many slopes (species)	-11404.31

Table S10. Deviance information criterion (DIC) values from hierarchical Bayesian multivariate phylogenetic regressions with both codon lengths and surface as response variables. The best-fit model DIC is in **bold**.

Model predictors	DIC mormoopids	DIC no mormoopids
Nucleotide length and log mass	-32863.55	-31123.06
Nucleotide length	-37342.32	-35448.98
Nucleotide length and log mass, mass slopes by animal/plant	**-37343.42**	-35446.31
Nucleotide length and log mass, length slopes by *OR* gene family, mass slopes by animal/plant	-37342.71	**-35451.30**

Table S11. Stepping-stone marginal likelihood of bayou reverse-jump Monte Carlo models relating surface to mass in log scale. The best-fit model is in **bold**.

Model	marginal likelihood
Single intercept, single slope	-47.6
**Many intercepts, single slope**	**-30.8**
Two intercepts, single slope	-45.8
Many intercepts, many slopes	-50.6

Table S12. Summary of best-fit bayou model of the allometry of olfactory epithelium surface area on body mass. Lower and upper correspond to 95% posterior high probability density intervals.

	**Mean**	**Lower**	**Upper**	**Effective Size**
Log-likelihood	-39.95	-48.44	-31.96	423
prior	-40.47	-59.08	-26.60	396
alpha	30.78	0.10	86.27	635
σ^2	52.27	0.12	148.15	568
Slope log(Mass)	0.81	0.21	1.36	102
Regime shifts	5	0	11	260
No. optima	6	1	12	260
Root optimum	1.82	-0.50	3.71	132

SI References

1. J. C. Uyeda, M. W. Pennell, E. T. Miller, R. Maia, C. R. Mcclain, The evolution of energetic scaling across the vertebrate Tree of Life. *Am. Nat.* **190**, 185–199 (2017).
2. Q. Martinez, *et al.*, Convergent evolution of an extreme dietary specialisation, the olfactory system of worm-eating rodents. *Sci. Rep.* **8**, 1–13 (2018).
3. M. A. Butler, A. A. King, Phylogenetic comparative analysis: A modeling approach for adaptive evolution. *Am. Nat.* **164**, 683–695 (2004).
4. D. Rojas, M. J. Pereira, C. Fonseca, L. M. Davalos, Eating down the food chain: generalism is not an evolutionary dead end for herbivores. *Ecol. Lett.* **21**, 402–410 (2018).
5. L. R. Yohe, *et al.*, Evaluating the performance of targeted sequence capture, RNA-Seq, and degenerate-primer PCR cloning for sequencing the largest mammalian multigene family. *Mol. Ecol. Resour.* **20**, 140–153 (2020).
6. L. R. Yohe, S. Hoffmann, A. Curtis, Vomeronasal and olfactory structures in bats revealed by diceCT clarify genetic evidence of function. *Front. Neuroanat.* **12**, 1–13 (2018).
